# Maintaining Outcomes of Internet-Delivered Cognitive-Behavioral Therapy for Depression: A Network Analysis of Follow-Up Effects

**DOI:** 10.3389/fpsyt.2021.598317

**Published:** 2021-04-20

**Authors:** Tim Kaiser, Lynn Boschloo, Thomas Berger, Björn Meyer, Christina Späth-Nellissen, Johanna Schröder, Fritz Hohagen, Steffen Moritz, Jan Philipp Klein

**Affiliations:** ^1^Department of Psychology, University of Salzburg, Salzburg, Austria; ^2^Department of Psychology, University of Greifswald, Greifswald, Germany; ^3^Faculty of Behavioural and Movement Sciences, Clinical Psychology, Vrije Universiteit Amsterdam, Amsterdam, Netherlands; ^4^Department of Psychology, Bern University, Bern, Switzerland; ^5^GAIA Hamburg, Hamburg, Germany; ^6^Department of Psychiatry, Lübeck University, Lübeck, Germany; ^7^Department of Psychiatry and Psychotherapy, University Medical Center Hamburg Eppendorf, Hamburg, Germany

**Keywords:** depression, network analysis, maintenance, internet interventions, health-related quality of life

## Abstract

**Background:** Depression is a highly prevalent mental disorder, but only a fraction of those affected receive evidence-based treatments. Recently, Internet-based interventions were introduced as an efficacious and cost-effective approach. However, even though depression is a heterogenous construct, effects of treatments have mostly been determined using aggregated symptom scores. This carries the risk of concealing important effects and working mechanisms of those treatments.

**Methods:** In this study, we analyze outcome and long-term follow-up data from the EVIDENT study, a large (*N* = 1,013) randomized-controlled trial comparing an Internet intervention for depression (Deprexis) with care as usual. We use Network Intervention Analysis to examine the symptom-specific effects of the intervention. Using data from intermediary and long-term assessments that have been conducted over 36 months, we intend to reveal how the treatment effects unfold sequentially and are maintained.

**Results:** Item-level analysis showed that scale-level effects can be explained by small item-level effects on most depressive symptoms at all points of assessment. Higher scores on these items at baseline predicted overall symptom reduction throughout the whole assessment period. Network intervention analysis offered insights into potential working mechanisms: while deprexis directly affected certain symptoms of depression (e.g., worthlessness and fatigue) and certain aspects of the quality of life (e.g., overall impairment through emotional problems), other domains were affected indirectly (e.g., depressed mood and concentration as well as activity level). The configuration of direct and indirect effects replicates previous findings from another study examining the same intervention.

**Conclusions:** Internet interventions for depression are not only effective in the short term, but also exert long-term effects. Their effects are likely to affect only a small subset of problems. Patients reporting these problems are likely to benefit more from the intervention. Future studies on online interventions should examine symptom-specific effects as they potentially reveal the potential of treatment tailoring.

**Clinical Trial Registration:** ClinicalTrials.gov, Identifier: NCT02178631.

## Background

### Internet-Delivered Psychotherapy

Depression has become one of the greatest challenges to public health, especially in Western, industrialized societies. An increasing number of persons with mental health problems seek treatment. However, structural barriers prevent many of those affected from getting the best help possible (1, 2). Depending on the health care system, a major structural barrier results from financial reasons (treatment costs or lack of insurance coverage), lack of time for undergoing treatment, or lack of clarity about where to get an appointment. Patients' attitudes can also prevent them from getting treatment, mainly because mental health problems are expected to improve without treatment by a mental health professional, but also due to fear of stigmatization or involuntary hospitalization (3).

As a result, the need for evidence-based and cost-effective treatments that are easy to disseminate becomes evident. Internet-delivered psychotherapy could have the potential to overcome the aforementioned barriers, as accessing them is easy and practically anonymous (4), while being cost-effective compared to care as usual (5). In-depth interview studies with patients show that internet-based treatment components also increase accessibility when blended with in-person treatment settings (6). For psychiatric and psychosomatic disorders, internet-delivered interventions show effects that are comparable to face-to-face treatments (7). Massoudi and colleagues showed that interned-delivered psychotherapy is moderately effective compared to waiting list control groups while small effects can still be observed when compared to care as usual (8). They also summarized four studies reporting cost-effectiveness, concluding that online interventions reduce healthcare costs by significant amounts. Internet-delivered psychotherapy can therefore be regarded as a useful addition to the mental health care system and could be implemented in stepped-care approaches as well as blended interventions in routine care (9, 10). In their review, Massoudi et al. found the most evidence for Internet-delivered cognitive-behavioral therapy (iCBT). iCBT can currently be considered the most promising alternative to traditional face-to-face approaches. Patients use specially designed secure websites or mobile applications over a specified period of time. Most of these programs consist of systematic presentation of therapy content through text, instructions for independent practice of learned techniques (“homework”), and accompanying materials such as videos or audio recordings. Some programs include contact to therapists via e-mail or video conferencing software, but many completely self-guided programs exist (11). While these analyses show promising results, there are cases in which internet-delivered psychotherapy shows small or unsatisfactory effects. A recent meta-analysis on iCBT for anxiety and depression in adolescents and young adults showed moderate effects for post-treatment symptom scores, but only small effects at follow-up (12). Generally, follow-up effects of iCBT tend to be small and non-significant. While it is possible that the effects of these interventions diminish after some time, another reason may also lie in the way symptoms are measured in most studies.

### Current Issues in the Measurement of Treatment Effects

In studies evaluating the effects of psychiatric and psychotherapeutic interventions, it is common practice to report mean differences. The reported mean values typically consist of the sum or average scores of psychometric scales to measure the severity of symptoms. However, Fried and Nesse (13) argued that symptoms of mental disorders should be analyzed on the item level, because important information is lost when using sum scores. They provided several examples for this in the example of depression. First, research on biomarkers for depression revealed that many of its biological correlates are symptom-specific (14). Second, many treatments are effective for specific symptoms only. For example, antidepressants were found to reduce depressive mood, anhedonia and feelings of worthlessness while their side-effects often mimic other symptoms like sleep problems, fatigue and suicidal ideation (15, 16). Psychotherapy might target different symptoms and could also have side-effects. Fournier et al. (17) found that cognitive psychotherapy reduced atypical-vegetative symptoms like hypersomnia, weight gain or changes in appetite. Bekhuis et al. (18) analyzed the effects of psychotherapy compared to psychotherapy combined with antidepressants and found that combined therapy was significantly more effective in reducing symptoms of feeling entrapped, emotional lability, worry, hopelessness, obsessive thoughts, blue mood, and feeling low in energy. Regarding side-effects, a qualitative analysis of a large sample found that patients receiving iCBT frequently report increases of anxiety, stress or insomnia (19). Thus, using sum scores could lead to inaccurate assessments of the efficacy of available treatments. Third, symptoms of depression are differentially associated with overall psychological functioning. Changes on the item level can be manifold but lead to the same changes on the scale level. Clinically, however, changes on a scale value can have various meanings. Using an example from a depression scale, a decrease in suicidality has completely different clinical implications than a change in eating behavior. The different arguments against the use of scale values were followed by an increase of research on symptom-specific intervention effects. For example, Hieronymus and colleagues (15) have shown that the apparent ineffectiveness of antidepressants in less severe depression is no longer detectable when examining the symptoms included in the 6-item Hamilton Depression Rating Scale. For depressed mood, feelings of guilt, impairment of work and loss of interest, psychomotor retardation, psychic anxiety, and general somatic symptoms, antidepressant effects were independent of baseline severity.

### Network Analysis

A promising method that is suitable for analyzing symptom-specific effects of psychological and psychiatric interventions, which has gained great popularity in recent years, is network analysis (20). When applied to psychopathology, mental disorders are treated as systems of interrelated symptoms. Typically, a correlation matrix for the items is calculated and transformed into a partial correlation matrix. Partial correlations indicate the pairwise relationship between two symptoms after possible confounding influences of other items in the network model have been removed. These partial correlations can be used, for example, to better understand the structure of the co-morbidity of mental disorders (21, 22).

The usefulness of the network approach is not limited to epidemiology but can also provide interesting insights for the therapy of mental disorders. In addition to symptoms, a binary “treatment” variable can be included in a network model. Symptoms that correlate negatively with this variable are directly affected by the intervention. We refer to this as “direct effects.” Symptoms that in turn correlate with the directly affected symptoms may also change, which we refer to as “indirect effects.”

Following this approach, Boschloo et al. (23) performed a network analysis and found that Deprexis directly targets a subset of depressive symptoms (feelings of guilt, concentration problems, fatigue and sleep problems) and that participants with high scores on these symptoms benefit more from the intervention. Blanken and colleagues (24) introduced a new network-based method to investigate such symptom-specific treatment effects: Network Intervention Analysis (NIA). In their study of patients suffering from insomnia, network models were used to analyze the sequential effects of an internet-delivered cognitive-behavioral intervention for insomnia compared to a waiting list control group over the course of several points of assessment. They could not show that the intervention primarily reduced insomnia symptoms and that these effects followed a certain temporal order. First, early morning awakening was reduced in the first week, followed by suicidal thoughts an the second week and difficulty maintaining sleep in week 3 and dissatisfaction with sleep in week 4. Depressive symptoms correlated with these symptoms reduced as well, suggesting indirect effects that result from an improvement of insomnia symptoms caused by the intervention.

Only recently has this field of research been looking at the effects of interventions beyond symptoms commonly associated with depression. A study by Cervin et al. (25) used network intervention analysis to study the symptom-specific effects of congnitive-behavioral therapy, antidepressant medication and their combination in pediatric anxiety disorders. They could show that, in addition to symptom reduction, all treatments achieved their effects also by a reduction of family interference and avoidant behavior. NIA therefore offers important insights into the working mechanisms of psychiatric treatment that would be concealed if using scale values to determine treatment effects.

### The Current Study

The analysis presented here is based on the EVIDENT study, a large randomized controlled trial on the effects of an online cognitive-behavioral intervention for depression (Deprexis). Symptom-specific effects of Deprexis were already studied using data from this study (23) and in another data set (26). However, only depressive symptoms directly after the intervention were considered. Thus, we will include a measure of health-related quality of life in our analysis, which will potentially reveal treatment effects that go beyond symptom reduction. In addition, while Klein et al. (27) reported that Deprexis continues to show small but significant long-term scale-level effects until up to 1 year, symptom-level effects in the follow-up period have not yet been studied.

In summary, the goals of this study were two-fold: first, we were interested in the direct and indirect effects of this intervention both on depressive symptoms as well as health-related quality of life. Second, our goal was to examine symptom-level effects over an extended period of time of up to 12 months. Based on previous network intervention analysis studies, we hypothesized that (a) Deprexis usage is linked to a reduction to a limited set of symptoms, (b) that a significant portion of the Deprexis effect is expressed indirecty and (c) that direct treatment effects become less pronounced in the follow-up period while changes in item means remain relatively stable.

## Methods

### Trial Design and Participants

We analyzed data from the “Effectiveness of Internet-based Depression Treatment” (EVIDENT) trial (28). EVIDENT was a large (*N* = 1,013) multicenter randomized controlled trial comparing an internet-delivered cognitive-behavioral intervention (Deprexis) with care as usual (CAU) for mild to moderate depressive symptoms. Patients scoring between 5 and 14 on the Patient Health Questionnaire-9 [PHQ-9 (29)] were included in the study. Participants were randomized equally to one of those conditions. Participants of this trial were free to use any form of treatment, including medication and psychotherapy. The treatment group received access to the Internet intervention in addition to their usual treatment. The internet-delivered cognitive-behavioral intervention “Deprexis” is a 12-week individually-tailored self-help programme based on cognitive-behavioral therapy. It consists of 10 modules covering a variety of techniques, like cognitive restructuring, behavioral activation, acceptance, mindfulness exercises, problem solving. Deprexis can be used with or without guidance by a mental health professional. In the EVIDENT trial, participants with mild depressive symptoms [PHQ-9 sum score 5 to 9 (30)] received the unguided version, while participants with moderate symptoms (PHQ-9 sum score 10 to 14) were contacted once a week by a trained supporter via e-mail. After the 12-month randomized-controlled trial (RCT) period, the CAU group had access to Deprexis as well. A more detailed description of the Deprexis programme is given by Meyer et al. (31).

**Figure 1:**
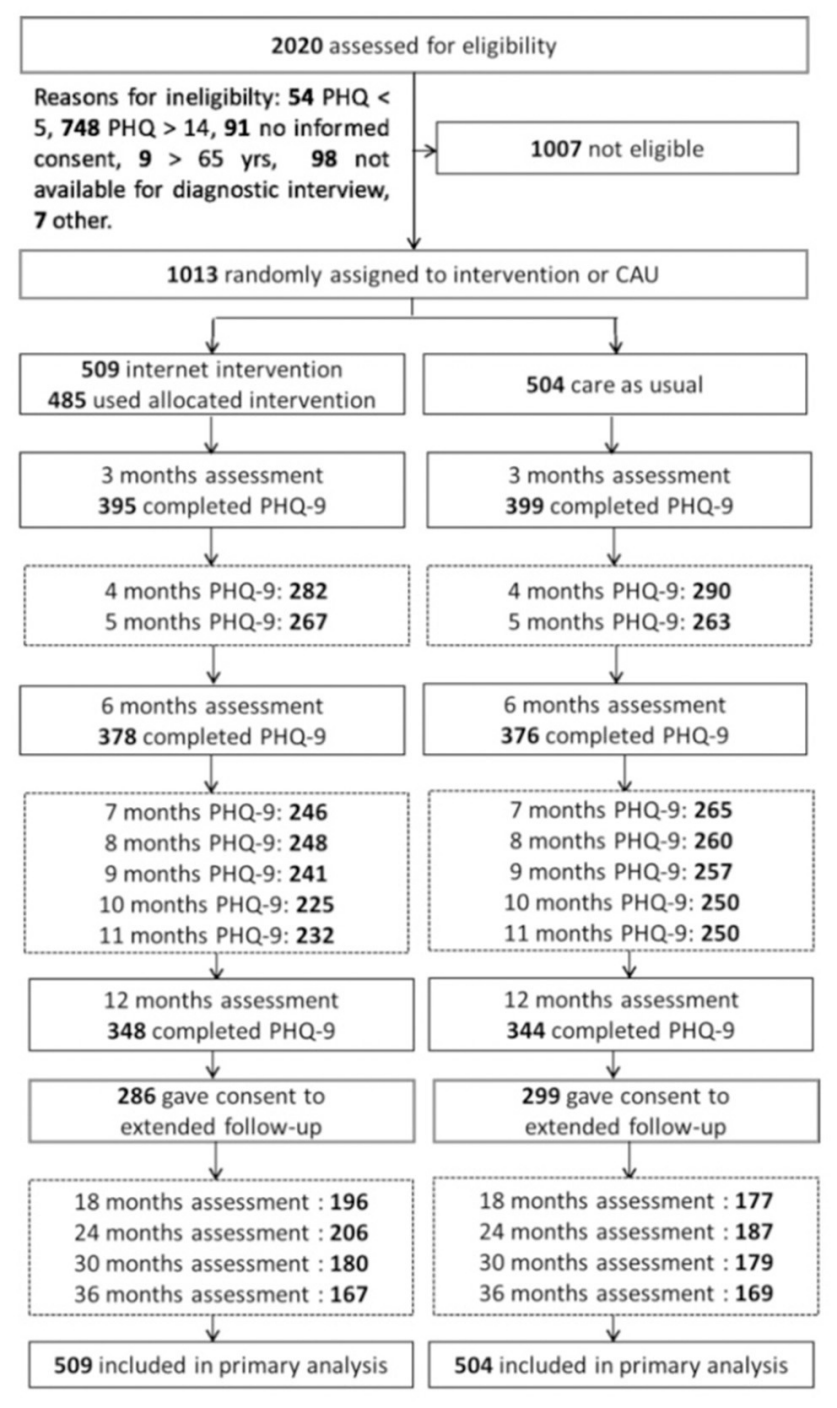
Study flowchart of the EVIDENT trial.

Depression symptoms and overall impairment were measured before trial onset (baseline), directly after the trial (post treatment) and over a follow-up period. Full assessments were conducted at three, 6 and 12 months after randomization. Additionally, monthly assessments of depressive symptoms were conducted between the post-assessment and the twelve-months follow-up. Then, an extended follow-up period of 18, 24, 30, and 36 months was offered to participants. In this period, the control group also had access to the Deprexis treatment. Figure 1 shows the study flow chart and sample sizes. While the EVIDENT trial conducted intention-to-treat analyses, we analyzed only the available data of every assessment. Sample sizes for all assessments included in this study are specified in Table 1.

**Table 1:** Overview of assessments, sample sizes, between-group standardized mean differences (Cohen's *d*) for main assessments and intermediary PHQ-9 assessments, including 95% confidence intervals.

**Timing of the assessment**	**PHQ-9**	**SF-12 mental**	**SF-12 physical**
3 months (post; *N* = 794)	−0.40 [−0.54; −0.26]	−0.45 [−0.30; −0.16]	−0.08 [−0.22; 0.06]
4 months (*N* = 572)	−0.44 [−0.61; −0.28]	-	-
5 months (*N* = 530)	−0.45 [−0.62; −0.27]	-	-
6 months (follow-up; *N* = 754)	−0.36 [−0.50; −0.21]	−0.21 [−0.35; −0.06]	−0.04 [−0.18; 0.11]
7 months (*N* = 511)	−0.32 [−0.49; −0.14]	-	-
8 months (*N* = 508)	−0.28 [−0.46; −0.11]	-	-
9 months (*N* = 498)	−0.23 [−0.41; −0.05]	-	-
10 months (*N* = 475)	−0.17 [−0.35; 0.01]	-	-
11 months (*N* = 482)	−0.11 [−0.29; 0.06]	-	-
12 months (follow-up; *N* = 692)	−0.27 [−0.43; −0.11]	−0.24 [−0.40; −0.07]	−0.08 [−0.24; 0.09]
18 Months (follow-up^*^; *N* = 373)	−0.14 [−0.35; 0.06]	−0.08 [−0.29; 0.13]	−0.17 [−0.38; 0.04]
24 Months (follow-up^*^; *N* = 393)	−0.14 [−0.34; 0.06]	−0.16 [−0.36; −0.04]	0.06 [−0.14; 0.26]
30 Months (follow-up^*^; *N* = 359)	−0.06 [−0.26; 0.15]	−0.01 [−0.22; 0.20]	0.08 [−0.22; 0.20]
36 Months (follow-up^*^; *N* = 336)	−0.03 [−0.18; 0.25]	0.02 [−0.19; 0.24]	0.14 [−0.08; 0.35]

*The SF-12 is not available for the monthly assessments. Negative values indicate lower scores for the Deprexis group compared to the CAU group*.

* *: participants from the CAU group had access to the intervention at this point*.

### Instruments

The nine-item Patient Health Questionnaire (PHQ-9) (29) was used as a primary outcome measure. The PHQ-9 is a relatively short, but highly reliable and well-validated self-report questionnaire for depression severity. In a validation study on the German general population, it was shown to have a one-factor structure and a high internal consistency (Cronbach alpha:.88).

The “Short Form-12” (SF-12) (32) was included to measure the perceived health status. It includes 12 items assessing the impact of impairment by physiological and psychological problems on health-related quality of life. The SF-12 has an acceptable internal consistency (Cronbach alpha:.89 for physical health,.76 for mental health) and was shown to be predictive of various health conditions (32).

### Statistical Analysis

In order to analyze the symptom-specific effects of the treatment, we used Network Intervention Analysis (24). We closely followed the proposed method by Blanken and colleagues (24), using Mixed Graphical Models (MGM) to estimate network models. For each assessment, we estimated a LASSO-regularized network that included the psychometric scales as well as a binary treatment allocation variable. Regularization methods like LASSO reduce the occurrence of false-positive edges in network models (33), thus minimizing spurious findings while increasing the interpretability of networks. The LASSO tuning parameter was selected using 10-fold cross-validation. We included all symptom items of the PHQ-9 and the SF-12 as continuous variables and added a binary treatment allocation variable (0: care as usual, 1: Deprexis intervention). The SF-12 included four yes/no items that were also treated as categorical binary variables. Because the CAU group got access to Deprexis after the 12-month follow-up, the extended follow-up assessments were excluded from network intervention analysis. If not stated otherwise, all analyses use the available data and missing data were removed using listwise deletion.

We used the resampling function implemented in the MGM package to conduct a bootstrap analysis of network edge stability. For every network model, we drew 100 bootstrap samples for which we fitted the models. We then plotted the sampling distribution for every edge weight. The plots show the number of times an edge was estimated to be non-zero when resampling, as well as the 5 and 95% quantiles of the estimates. We will report the stability estimate (i.e., the percentage of bootstrap runs in which the edge was estimated as being non-zero) for the reported edge weights. For example, a stability of 98% indicates that the edge was found to be non-zero in 98 of 100 bootstrap runs. Full result plots for the resampling procedure of every model are available in the online supplement.

#### Graphical Representation

We used the R package qgraph (34) to plot the network models. In these plots, the nodes represent the symptoms and treatment allocation, while the edges represent partial correlations between nodes. Edges can be green, indicating a positive correlation between two nodes, or red, indicating a negative correlation. The thickness of edges is proportional to the strength of the correlation. For example, red connection between the Treatment node and a symptom indicates that a reduction of this symptom can be explained by using Deprexis. The node size changes if a mean score changes relative to its baseline over and above changes in the CAU group. Graph layout was done using a fixed, three-layer structure: on top, the “intervention” variable was placed. Below, items directly affected by the treatment are positioned. The third layer contains all other items. To ease the interpretation of successive graphs, the layout from the baseline graph was used for all other graphs as well.

#### Estimation of Direct and Indirect Effects

Network models can be analyzed by calculating a number of node centrality measures (33). These measures are used to estimate the influence of single nodes in a model on other nodes. For example, by summing the absolute edge weights of one node, its “strength” can be calculated. “Betweenness” indicates how often one node lies on the shortest path between two other nodes. To determine direct as well as indirect effects of the intervention, we used the centrality measure “bridge expected influence” (BEI) (35). This measure is defined as the sum of signed edge weights that connect nodes from two predefined communities. We defined the treatment allocation variable as one “community” and PHQ-9 and SF-12 items as another. Thus, the direct effect of Deprexis usage on the measured symptoms is the BEI of the treatment allocation variable. The BEIs of the symptom nodes show how the overall effect can be broken down into symptom-specific effects. The “two-step BEI” can be computed by taking into account the influence of nodes affected by the intervention on other nodes. For example, if the treatment reduces “depressive mood” and the “depressive mood” item is correlated with “suicidality,” the two-step BEI would include the connections between those symptoms because a reduction of “depressive mood” is likely to reduce “suicidality” as well. Thus, the “two-step BEI” can be used as an estimate of additional indirect treatment effects.

#### Visualizing Treatment Effects on Symptom Severity

As proposed by Blanken et al. (24), we standardized item values at each assessment to the baseline value and subtracted the standardized differences of the treatment group from those of the control group. This way, we can visualize the symptom reduction that can be attributed to the intervention. Smaller nodes in the network plots are those most affected by the intervention.

#### Predicting Treatment Effects From Baseline Score Profile

Similar to Boschloo et al. (23), we were interested in the predictive utility of item scores affected by the treatment in the network models. Thus, we calculated a baseline severity indicator by averaging item scores of those items affected by the treatment in the “post” model. As a control, we calculated another index consisting of all items not directly affected by the treatment. We then correlated this indicator with symptom reductions at all assessment points.

## Results

### Outcome

The between-group effect sizes on the PHQ-9 and the two subscales of the SF-12 are summarized in Table 1. It shows that the mean mental health symptom burden is stable for up to 12 months after the start of the study. These effects were not found in the extended follow-up period in which the CAU group also had access to Deprexis.

### Network Intervention Analysis

Despite the modest effects at the scale level, it is possible that the long-term effects can only be seen on certain items. In order to gain a more detailed insight into the effects of deprexis and to be able to distinguish indirect from direct effects, NIA was used. Some BEI values are used for interpreting treatment effects. See Appendix A5 for a full summary of BEI values for each item and assessment. For the sake of reproducibility, correlation matrices of all network models are provided in the online supplement.

**Figure 2:**
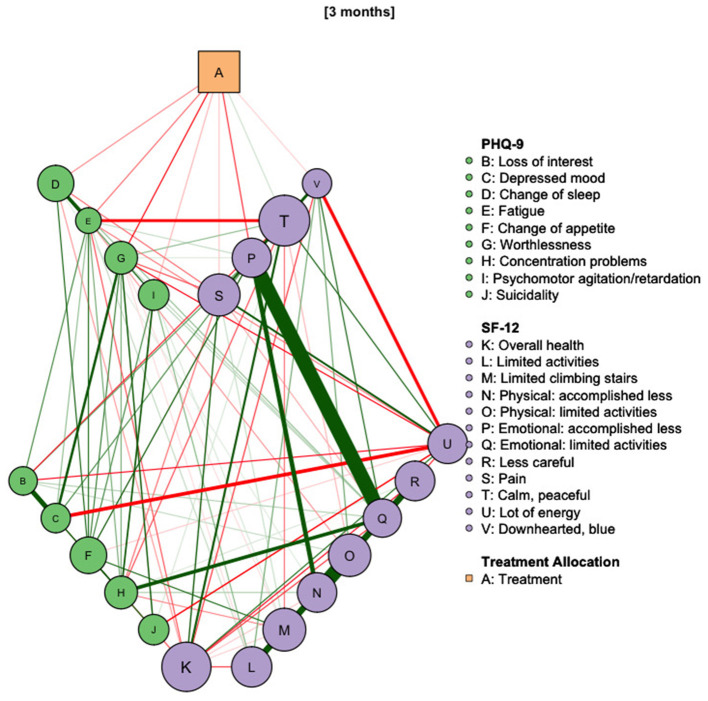
Network diagram of treatment allocation, depressive symptoms and health-related quality of life at post-treatment assessment. Red edges indicate negative correlations, green edges indicate positive ones. Smaller nodes have greater pre-post mean reductions compared to the control group. Nodes directly affected by the treatment are arranged in the first line. *N* = 794.

The network model revealed that the effect of Deprexis was directed at a subset of symptoms. As depicted in Figure 2, using Deprexis directly reduced a subset of seven item scores: “worthlessness” (BEI: −0.12, stability of the edge, measured by percentage of non-zero estimates in bootstrap runs: 100%), “accomplished less because of emotional problems” (BEI: −0.09, stability: 74%), “fatigue” (−0.06, stability: 82%), “change of sleep” (−0.05, stability: 74%), “psychomotor agitation” (−0.03, stability: 71%), “pain” (−0.02, stability: 72%) and “downhearted, blue” (−0.02, stability: 52%). The item “Calm, peaceful” showed a slight increase that can directly be explained by using Deprexis (0.02, stability: 62%). Additionally, indirect treatment effects can be observed. For example, Deprexis use was most strongly associated with reduced “worthlessness,” which is positively correlated with “depressed mood.” This suggests that reductions of “depressed mood” can be explained by the direct reduction of “worthlessness.” A reduction of “fatigue” on the other hand will lead to an increase in “calm peaceful,” as indicated by a negative connection between these two items.

The BEI of the treatment node was −0.36 in the first step and −0.62 in the second step, suggesting that a significant proportion of the treatment effect is indirect.

**Figure 3:**
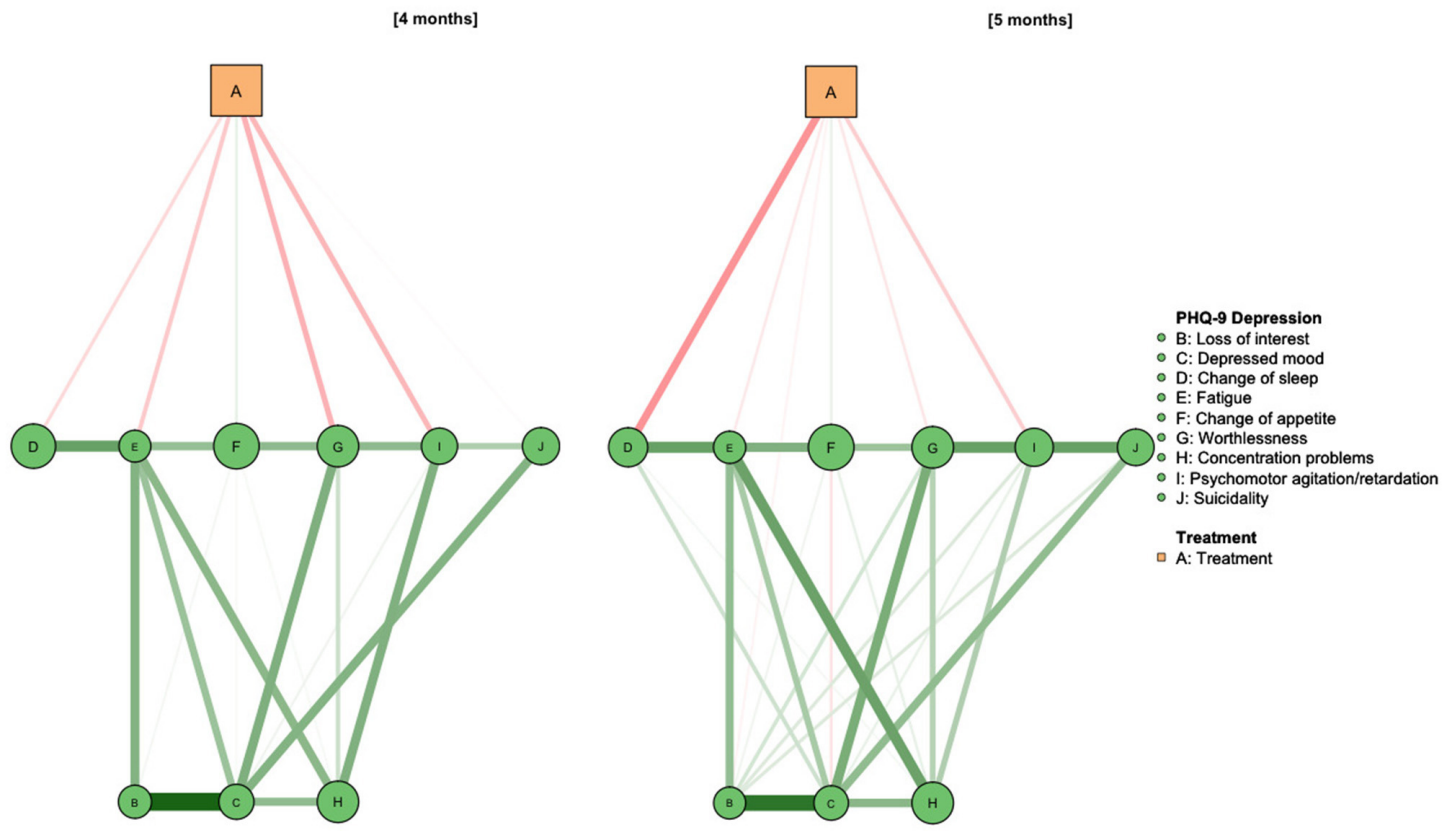
Network diagram of treatment allocation, depressive symptoms and health-related quality of life at 4 and 5 months after study onset. Ns = 572 (4 months) and 530 (5 months).

**Figure 4:**
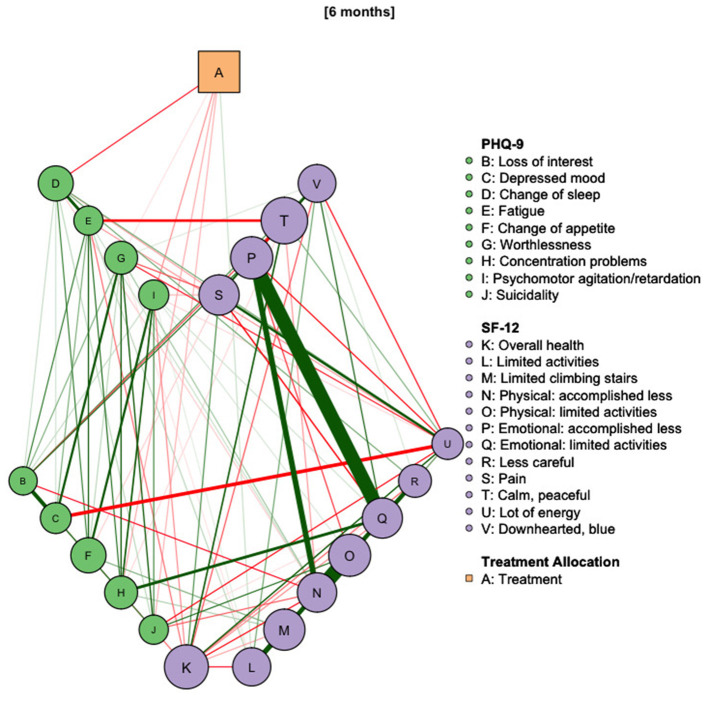
Network diagram of treatment allocation, depressive symptoms and health-related quality of life at 6 months after study onset. *N* = 754.

As depicted in Figure 3, network models for the intermediary PHQ-9 assessments at 4 and 5 months again revealed the association of Deprexis treatment with “Worthlessness” (stability: 98%), “Psychomotor agitation” (stability: 95%), “Fatigue” (91%) and “Change of sleep” (stability: 82%). In the 5 months-assessment, treatment was most strongly associated with “Change of sleep” (stability: 99%), while reductions of other symptoms, as well as the overall network structure, remained relatively stable. First- and second-step BEIs of the treatment variable were −0.34/−0.62 after 4 months and −0.32/−0.39 after 5 months. As shown in Figure 4, this effect was also observed after 6 months. At this point, Deprexis usage was associated with “Change of sleep” (BEI: −0.12, stability: 98%), “Worthlessness” (−0.01, stability: 61%), “Concentration problems” (−0.04, stability: 73%), “Psychomotor agitation” (−0.05, stability: 86%), and “Suicidality” (−0.04, stability: 67%).

**Figure 5:**
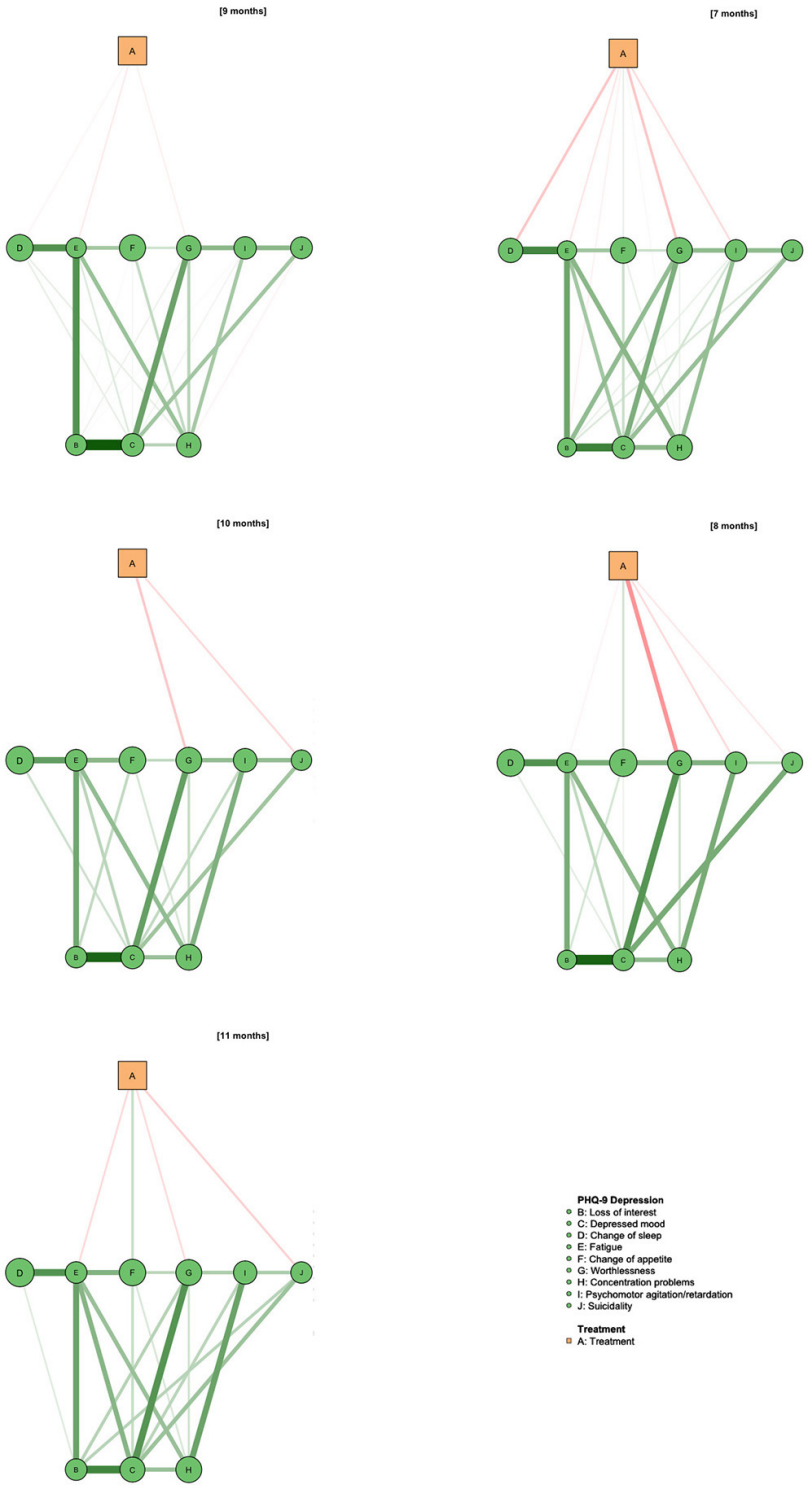
Intermediary assessments for the 7 to 11-month follow-up assessments. *N* = 511, 508, 498, 475, and 482, respectively.

**Figure 6:**
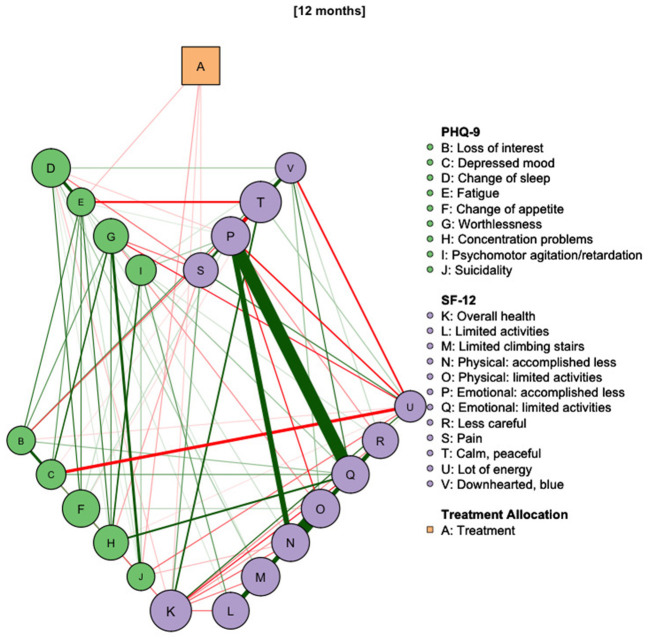
Network diagram of treatment allocation, depressive symptoms and health-related quality of life at 12 months after study onset. *N* = 637.

The treatment effects decrease continuously in the course of the further follow-up time. This can be seen in Figure 5 and is indicated by the drop in BEI at 7 months (−0.24) to −0.09 at 11 months. The last follow-up assessment we analyzed was conducted at 12 months and is depicted in Figure 6. Here, effects on the PHQ-9 were comparable to the previous assessments (treatment BEI: −0.10) and the overall effects can be explained by small effects on “Fatigue” (BEI: −0.05, stability: 67%) and “Suicidality” (BEI: −0.03, stability: 73%). When taking account indirect effects, the BEI of the treatment variable increases to −0.28.

### Prediction of Treatment Outcomes and Stability by Baseline Severity Indicator

As shown in Table 2, the baseline severity indicator was significantly correlated with greater PHQ-9 symptom reductions in the Deprexis group, with the exception of the 6 months-assessment. In the CAU group, the indicator was correlated with stronger outcomes in the 36 months-assessment. The control indicator was not significantly associated with outcomes at any time point (All |r| < 0.16, *p* >0.18).

**Table 2:** Correlations of baseline severity indicator with symptom reductions at different time points.

**Time (months)**	**Deprexis**	**CAU**
3	−0.23^*^	−0.07
6	−0.13	−0.07
12	−0.21^*^	−0.15
18	−0.26^*^	−0.15
24	−0.25^*^	−0.18
30	−0.35^*^	−0.17
36	−0.24^*^	−0.35^*^

* *, Holm-adjusted p < 0.05*.

## Discussion

In this study, we applied a novel approach to evaluating long-term treatment efficacy to a data set from the EVIDENT trial (EffectiVeness of Internet- based DEpressioN Treatment) which has examined the effectiveness of “Deprexis,” an online intervention for depression. This is the first study using Network Intervention Analysis for studying long-term follow-up effects of internet-delivered treatments of depression. Our findings can be summarized as follows:

First, effects of Deprexis in addition to CAU are likely to result by affecting activity patterns (indicated by direct treatment effects on “fatigue” and “psychomotor agitation/retardation”), sleep behavior (“change of sleep”) and depressive cognitions (“worthlessness”).

Second, patients who score higher on the directly affected items (“worthlessness,” “accomplished less because of emotional problems,” “fatigue,” “change of sleep,” “psychomotor agitation,” “pain,” “downhearted, blue” and “Calm, peaceful”) at baseline profit more from Deprexis throughout the whole study period. This could indicate a patient subtype that benefits particularly from Deprexis.

Third, a large portion of treatment effects can be better explained by changes in other symptoms than by assignment to the treatment group. We examined this relation of direct to indirect treatment effect size by comparing one-step and two-step expected influence values. This means that Deprexis tackles only a small subset of problems but is successful in reducing many other symptoms as a secondary effect. For example, “Depressed mood” was significantly correlated with “Worthlessness” and also decreased after treatment. Similarly, “Fatigue” was directly affected and correlated with “Loss of interest” as well as “Change of appetite,” both of which showed decreases in the treatment group.

Fourth, Deprexis usage affects health-related quality of life by reducing depressive feelings, increasing feelings of calmness, reducing functional impairment by emotional problems, and pain.

These results presented increase the understanding of the effects of online interventions on depression by also evaluating follow-up data. They also reveal how changes at the symptom level affect the health-related quality of life.

### Scale and Item-Level Treatment Effects

Small to moderate between-group effects were found on the scale level until up to 12 months after study onset for depressive symptoms measured by the PHQ-9 and for mental health related quality of life measured by the SF-12. No effects were found for the physical health related quality of life. In the extended period, CAU participants could also access Deprexis. During this period, group differences on the scale level disappeared completely, indicating that even this delayed treatment with Deprexis had at least some effects for the CAU group as well.

At the item level, it was found that different PHQ-9 items reacted differently to the treatment. Between-group effects were smaller and more transient for some items (e.g., “Change of appetite,” “Change of sleep,” “Concentration problems,” and “Worthlessness”) than for others (e.g., depressed mood, fatigue, loss of interest and suicidality). It is noteworthy that the strongest and longest lasting effect could be demonstrated for the suicidality item. Given the high scalability of online interventions like Deprexis, even the modest effects observed in this study can be relevant.

Interestingly, participants scoring higher on the items affected by Deprexis at the post assessment showed stronger treatment outcomes throughout the whole trial period, including the follow-up period, a finding that could have implications for further personalization of this intervention. For example, while patients reporting higher values for “Worthlessness”, “Fatigue”, “Psychomotor agitation” and “Change of sleep” might be directed to the standard version of Deprexis, while a modified version of the treatment could be developed for patients reporting higher scores on the other items. This modified version might be specifically tailored to address problems that we did not find to be directly affected by Deprexis treatment. Those symptoms include “Loss of interest” and “Lot of energy” from the SF-12, so the modification could be more effective by emphasizing techniques that focus on behavioral activation (36) more strongly. The fact that the baseline severity index also correlated with the effect in the CAU group at the 36 months assessment can possibly be explained by the fact that the CAU group could access Deprexis after the 12 months follow-up.

### Network Analysis

By using network analysis and including a treatment variable, we were able to isolate direct and indirect effects of Deprexis. In contrast to the mere analysis of changes in mean scores, this type of analysis goes beyond looking at symptom reduction and can provide information about possible mechanisms of action. Although connections in the network and symptom reduction correlate at the item level, not every item that showed a decreased mean value must also have a connection in the network. Instead, the items tend to have a connection to the treatment, the reduction of which cannot be explained by the reduction of other symptoms. The strong direct effect on self-devaluating cognitions (measured by the PHQ-9 item “Worthlessness”) is expected from cognitive-behavioral therapy, as changing depressive cognitions are the main focus of CBT (37). Similarly, reductions in “Fatigue” and “Accomplished less because of emotional problems” could be in line with behavioral activation and reduction of avoidant behavior, both of which are well-established working mechanisms in CBT for depression (38).

Indirect (second-step) treatment effects were more diverse and encompassed most of the PHQ-9 items. Since we were only able to distinguish Deprexis and CAU group, we can only speculate which specific ingredients of the intervention led to these effects. Assuming that “worthlessness” predominantly captures depressive cognitions, the effect in the network model could be explained by exercises in cognitive reappraisal.

While the symptom-specific effects on the depression questionnaire PHQ-9 were clearly visible in the analysis, there were less pronounced effects for the general health questionnaire SF-12. Deprexis users reported being constrained by emotional problems less frequently, but other symptoms were not directly linked to the treatment. Indirect effects also revealed negative associations between Deprexis usage and SF-12 items reflecting depressive symptoms (“downhearted, blue”). Since BEI only involves up to two “steps” in the network, treatment effects may be more indirect or caused by variables not included in our model. However, given our results it can be assumed that Deprexis works mainly by reducing depressive symptoms directly, which leads to a subsequent increase of quality of life. This finding is expected, as the PHQ-9 was found to be highly correlated with most items of the SF-12 (39). Also, there were strong intercorrelations of SF-12 items that are most likely due to the structure of the questionnaire. For example, items asking if a participant “Accomplished less than they would like” and “Were limited in the kind of work or other activities” (items N and O in network graphs), a very high correlation is almost guaranteed, especially with a binary item format.

By applying the network intervention analysis approach to follow-up data, we identified symptoms responsible for the maintenance of treatment effects over 1 year after study onset. Interestingly, those symptoms were different to those associated with treatment in the first assessment. It could be speculated that effects on this aspect of depression need several months to unfold although further studies including more intense repeated assessments are needed to substantiate this.

### Limitations

Some limitations to our findings should be considered. Especially in the follow-up period, there was a considerable number of drop-out cases. This could have led to less accurate estimates of network edges and false-positive results. Since this study is a secondary data analysis, no a priori simulation study for power calculation was possible. However, as our bootstrap analysis shows, many edges estimated for treatment effects show imperfect stability, possibly reducing the confidence once should put in these effects. On the other hand, no agreed-upon cutoff values for satisfactory edge stability in network intervention analysis exist and the stability of treatment effect edges obtained in this study are comparable and often surpass those reported by Blanken et al. (24).

Regarding treatment effects, we decided to analyze complete cases because the network modeling approach we chose does not support incomplete data. This could have led to inaccurate effect size estimates. In fact, effect sizes from the previously reported intention-to-treat analysis by Klein et al. (27) were lower and less stable at follow-up.

The attribution of treatment effects observed in our sample is somewhat complicated due to a number of factors. Because participants were able to continue with their current psychiatric or psychotherapeutic treatment, a part of the effect might be attributable to these ongoing treatments. Subgroup analyses conducted in the original study (28) showed that the treatment effect of DEPREXIS was smaller in those patient groups. However, the proportion of patients receiving treatments elsewhere was the same in both groups by randomization, allowing the cautious conclusion that the impact of these treatments was not significantly greater in either group. While higher symptom severity normally predicts slightly larger effects in low-intensity interventions (40), this moderator is likely have been washed out because individual treatments were started long before participating in the EVIDENT study. Thus, including participants that already underwent other treatments is likely to have led to an underestimation of treatment effects. On the other hand, participants with higher PHQ-9 scores (sum score of 10 to 14) were contacted by a supporter once per week, which could have led to increased effects for this group. Because symptom severity and access to e-mail support are confounded, it is hard to separate the influences of these two conditions. Future studies should consider randomizing supporter conditions to make this possible.

Compared to the work by Blanken et al. (24), we could not study the effects during the ongoing treatment because there were no assessments during that time. The processual nature of therapeutic interventions is lost in cross-sectional designs because intraindividual variation are not captured (41, 42). This requires study designs with high frequency measurements of the therapeutic process. For example, Santos et al. (38) used repeated measures during behavioral activation-focused residential treatment to show that the extent of behavioral activation is in fact associated with treatment outcomes. Future studies on the working mechanisms of online interventions should include adequate measures of intraindividual variation.

### Outlook

Future studies could extend the network intervention analysis approach to studies comparing two or more treatment approaches. This way, important information about the symptom-specific effects of different treatments could be uncovered, possibly leading to personalized treatment recommendations. Ideally, researchers considering using this approach should design their clinical studies in a way that NIA can be carried out adequately. This includes adding ongoing symptom assessments to the design of clinical studies so that the unfolding of treatment effects can be observed during interventions. Also, a priori statistical power calculations should include network models in order to guarantee stable estimates.

### Conclusion

Online interventions can help participants to manage their symptoms more effectively. In the case of Deprexis, this is accomplished most likely by reducing depressive thoughts and fatigue. Network intervention analysis is a promising tool to help clinical psychologists to design and evaluate interventions that lead to a broadening of knowledge about treatment effects and, thus, to greater benefits for participants.

## Data Availability Statement

The data analyzed in this study is subject to the following licenses/restrictions: The data that support the findings are not publicly available, as the publication of the collected primary data is not covered by the informed consent. For the sake of reproducibility, correlation matrices of all network models are provided in the online supplement. Requests to access these datasets should be directed to philipp.klein@uksh.de.

## Ethics Statement

The studies involving human participants were reviewed and approved by The article is a secondary data analysis of the EVIDENT trial. This trial has been approved by the Ethics Committee of the German Psychological Association (DGPs, reference number SM 04_2012). The patients/participants provided their written informed consent to participate in this study.

## Author Contributions

TK developed the concept of the paper, the first draft, the final manuscript and performed all steps of the data evaluation. LB has made key methodological and theoretical contributions as well as contributing to the revision of the manuscript. TB made a decisive contribution to the revision of the manuscript. BM, CS-N, JS, FH, and SM all provided important remarks on the first draft. JK provided important theoretical and methodological contributions and coordinated communications between co-authors. All authors contributed to the article and approved the submitted version.

## Conflict of Interest

JK received funding for clinical trials (German Federal Ministry of Health, Servier—distributor of the internet intervention “Deprexis”), payments for presentations on internet interventions (Servier) and payments for workshops and books (Beltz, Elsevier, Hogrefe and Springer) on psychotherapy for chronic depression and on psychiatric emergencies. BM is employed as research director at GAIA AG, the company that developed, owns and operates the internet intervention “Deprexis.” The remaining authors declare that the research was conducted in the absence of any commercial or financial relationships that could be construed as a potential conflict of interest.

## References

[1] Stein MB Roy-Byrne PP Craske MG Campbell-Sills L Lang AJ Golinelli D. Quality of and patient satisfaction with primary health care for anxiety disorders. J Clin Psychiatry. (2011) 72:970–6. 10.4088/JCP.09m05626blu 21367351 PMC3111814

[2] Bartram M Stewart JM. Income-based inequities in access to psychotherapy and other mental health services in Canada and Australia. Health Policy. (2019) 123:45–50. 10.1016/j.healthpol.2018.10.011 30424886

[3] Sareen J Jagdeo A Cox B Clara I Have M Bolton S-L. Perceived barriers to mental health service utilization in the United States, Ontario, and the Netherlands. Psychiatric Services. (2007) 58:357–64. 10.1176/ps.2007.58.3.357 17325109

[4] Griffiths KM Christensen H. Review of randomised controlled trials of Internet interventions for mental disorders and related conditions. Clin Psychol. (2006) 10:16–29. 10.1080/13284200500378696

[5] Arnberg FK Linton SJ Hultcrantz M Heintz E Jonsson U. Internet-delivered psychological treatments for mood and anxiety disorders: a systematic review of their efficacy, safety, and cost-effectiveness. PLoS ONE. (2014) 9:e98118. 10.1371/journal.pone.0098118 24844847 PMC4028301

[6] Schuster R Sigl S Berger T Laireiter A-R. Patients' experiences of web- and mobile-assisted group therapy for depression and implications of the group setting: qualitative follow-up study. JMIR Mental Health. (2018) 5:e49. 10.2196/mental.9613 29997106 PMC6060305

[7] Carlbring P Andersson G Cuijpers P Riper H Hedman-Lagerlöf E. Internet-based vs. face-to-face cognitive behavior therapy for psychiatric and somatic disorders: an updated systematic review and meta-analysis. Cogn Behav Ther. (2018) 47:1–18. 10.1080/16506073.2017.1401115 29215315

[8] Massoudi B Holvast F Bockting CLH Burger H Blanker MH. The effectiveness and cost-effectiveness of e-health interventions for depression and anxiety in primary care: A systematic review and meta-analysis. J Affect Disord. (2019) 245:728–43. 10.1016/j.jad.2018.11.050 30447572

[9] Etzelmueller A Radkovsky A Hannig W Berking M Ebert DD. Patient's experience with blended video- and internet based cognitive behavioural therapy service in routine care. Internet Interv. (2018) 12:165–75. 10.1016/j.invent.2018.01.003 30135780 PMC6096318

[10] Vis C Kleiboer A Prior R Bønes E Cavallo M Clark SA. Implementing and up-scaling evidence-based eMental health in Europe: The study protocol for the MasterMind project. Internet Interv. (2015) 2:399–409. 10.1016/j.invent.2015.10.002

[11] Andersson G. Using the Internet to provide cognitive behaviour therapy. Behav Res Ther. (2009) 47:175–80. 10.1016/j.brat.2009.01.010 19230862

[12] Christ C Schouten MJ Blankers M van Schaik DJ Beekman AT Wisman MA. Internet and computer-based cognitive behavioral therapy for anxiety and depression in adolescents and young adults: systematic review and meta-analysis. J Med Internet Res. (2020) 22:e17831. 10.2196/17831 32673212 PMC7547394

[13] Fried EI Nesse RM. Depression sum-scores don't add up: why analyzing specific depression symptoms is essential. BMC Med. (2015) 13:72. 10.1186/s12916-015-0325-4 25879936 PMC4386095

[14] Fried EI von Stockert S Haslbeck JMB Lamers F Schoevers RA Penninx BWJH. Using network analysis to examine links between individual depressive symptoms, inflammatory markers, and covariates. Psychol Med. (2019) 50:1–9. 10.31234/osf.io/5mvz4 31615595

[15] Hieronymus F Lisinski A Nilsson S Eriksson E. Influence of baseline severity on the effects of SSRIs in depression: an item-based, patient-level *post-hoc* analysis. Lancet Psychiatry. (2019) 6:745–52. 10.1016/S2215-0366(19)30216-0 31303567

[16] Baldwin D. Essential considerations when choosing a modern antidepressant. Int J Psychiatry Clin Prac. (2003) 7(Suppl 1):3–8. 10.1080/13651500310000825 24937412

[17] Fournier JC DeRubeis RJ Hollon SD Gallop R Shelton RC Amsterdam JD. Differential change in specific depressive symptoms during antidepressant medication or cognitive therapy. Behav Res Ther. (2013) 51:392–8. 10.1016/j.brat.2013.03.010 23644038 PMC3711944

[18] Bekhuis E Schoevers R de Boer M Peen J Dekker J Van H. Symptom-specific effects of psychotherapy versus combined therapy in the treatment of mild to moderate depression: a network approach. PPS. (2018) 87:121–3. 10.1159/000486793 29495015 PMC5969070

[19] Rozental A Boettcher J Andersson G Schmidt B Carlbring P. Negative effects of internet interventions: a qualitative content analysis of patients' experiences with treatments delivered online. Cogn Behav Ther. (2015) 44:223–36. 10.1080/16506073.2015.1008033 25705924

[20] Borsboom D Cramer AOJ. Network analysis: an integrative approach to the structure of psychopathology. Ann Rev Clin Psychol. (2013) 9:91–121. 10.1146/annurev-clinpsy-050212-185608 23537483

[21] Fried EI van Borkulo CD Cramer AOJ Boschloo L Schoevers RA Borsboom D. Mental disorders as networks of problems: a review of recent insights. Soc Psychiatry Psychiatr Epidemiol. (2017) 52:1–10. 10.1007/s00127-016-1319-z 27921134 PMC5226976

[22] Bringmann LF Lemmens LHJM Huibers MJH Borsboom D Tuerlinckx F. Revealing the dynamic network structure of the Beck Depression Inventory-II. Psychol Med. (2015) 45:747–57. 10.1017/S0033291714001809 25191855

[23] Boschloo L Cuijpers P Karyotaki E Berger T Moritz S Meyer B. Symptom-specific effectiveness of an internet-based intervention in the treatment of mild to moderate depressive symptomatology: The potential of network estimation techniques. Behav Res Ther. (2019) 122:103440. 10.1016/j.brat.2019.103440 31542565

[24] Blanken TF Van Der Zweerde T Van Straten A Van Someren EJW Borsboom D Lancee J. Introducing network intervention analysis to investigate sequential, symptom-specific treatment effects: a demonstration in co-occurring insomnia and depression. Psychother Psychosom. (2019) 88:52–4. 10.1159/000495045 30625483 PMC6469840

[25] Cervin M Storch EA Piacentini J Birmaher B Compton SN Albano AM. Symptom-specific effects of cognitive-behavioral therapy, sertraline, and their combination in a large randomized controlled trial of pediatric anxiety disorders. J Child Psychol Psychiatry. (2020) 61:492–502. 10.1111/jcpp.13124 31471911

[26] Mullarkey MC Stein A Pearson R Beevers CG. Network analyses reveal which symptoms improve (or not) following an Internet intervention (Deprexis) for depression. Depression Anxiety. (2019) 37:115–24. 10.31234/osf.io/ca5dg 31710772 PMC6992506

[27] Klein JP Späth C Schröder J Meyer B Greiner W Hautzinger M. Time to remission from mild to moderate depressive symptoms: one year results from the EVIDENT-study, an RCT of an internet intervention for depression. Behav Res Ther. (2017) 97:154–62. 10.1016/j.brat.2017.07.013 28797829

[28] Klein JP Berger T Schröder J Späth C Meyer B Caspar F. Effects of a psychological internet intervention in the treatment of mild to moderate depressive symptoms: results of the EVIDENT study, a randomized controlled trial. Psychother Psychosom. (2016) 85:218–28. 10.1159/000445355 27230863 PMC8117387

[29] Kroenke K Spitzer RL. The PHQ-9: a new depression diagnostic and severity measure. Psychiatr Ann. (2002) 32:509–15. 10.3928/0048-5713-20020901-06

[30] Klein JP Berger T Schröder J Späth C Meyer B Caspar F. The EVIDENT-trial: protocol and rationale of a multicenter randomized controlled trial testing the effectiveness of an online-based psychological intervention. BMC Psychiatry. (2013) 13:239. 10.1186/1471-244X-13-239 24074299 PMC3850933

[31] Meyer B Berger T Caspar F Beevers C Andersson G Weiss M. Effectiveness of a novel integrative online treatment for depression (deprexis): randomized controlled trial. J Med Internet Res. (2009) 11:e15. 10.2196/jmir.1151 19632969 PMC2762808

[32] Ware JE Kosinski M Keller SD. A 12-item short-form health survey: construction of scales and preliminary tests of reliability and validity. Med Care. (1996) 34:220–33. 10.1097/00005650-199603000-00003 8628042

[33] Epskamp S Borsboom D Fried EI. Estimating psychological networks and their accuracy: a tutorial paper. Behav Res Methods. (2017) 50:195–212. 10.3758/s13428-017-0862-1 28342071 PMC5809547

[34] Epskamp S Cramer AOJ Waldorp LJ Schmittmann VD Borsboom D. qgraph: network visualizations of relationships in psychometric data. J Stat Softw. (2012) 48:1–18. 10.18637/jss.v048.i04

[35] Jones PJ Ma R McNally RJ. Bridge centrality: a network approach to understanding comorbidity. Multivariate Behav Res. (2017) 1–15. 10.1080/00273171.2019.1614898 31179765

[36] Huguet A Miller A Kisely S Rao S Saadat N McGrath PJ. A systematic review and meta-analysis on the efficacy of Internet-delivered behavioral activation. J Affect Disord. (2018) 235:27–38. 10.1016/j.jad.2018.02.073 29649708

[37] Beck AT Rush AJ editors. Cognitive Therapy of Depression. 13th ed. New York, NY: Guilford Press (1979). p. 425.

[38] Santos MM Ullman J Leonard RC Puspitasari AJ Cook J Riemann BC. Behavioral activation as a mechanism of change in residential treatment for mood problems: a growth curve model analysis. Behav Ther. (2019) 50:1087–97. 10.1016/j.beth.2019.05.004 31735244

[39] Kocalevent R-D Hinz A Brähler E. Standardization of the depression screener Patient Health Questionnaire (PHQ-9) in the general population. Gen Hosp Psychiatry. (2013) 35:551–5. 10.1016/j.genhosppsych.2013.04.006 23664569

[40] Bower P Kontopantelis E Sutton A Kendrick T Richards DA Gilbody S. Influence of initial severity of depression on effectiveness of low intensity interventions: meta-analysis of individual patient data. BMJ. (2013) 346:f540. 10.1136/bmj.f540 23444423 PMC3582703

[41] Molenaar PCM. On the necessity to use person-specific data analysis approaches in psychology. Eur J Dev Psychol. (2013) 10:29–39. 10.1080/17405629.2012.747435

[42] Fisher AJ Medaglia JD Jeronimus BF. Lack of group-to-individual generalizability is a threat to human subjects research. PNAS. (2018) 115:E6106–15. 10.1073/pnas.1711978115 29915059 PMC6142277

