# Supplementary material for: Maintaining Outcomes of Internet-Delivered Cognitive-Behavioral Therapy for Depression: A Network Analysis of Follow-Up Effects
Source: Front Psychiatry. 2021 Apr 20;12:598317. doi: 10.3389/fpsyt.2021.598317 (PMC8095668; doi:10.3389/fpsyt.2021.598317)
Supplement: Supplementary file 1 [file Table_1.docx]

| Item | 3 Months | 6 Months | 12 Months | 18 Months* | 24 Months* | 30 Months* | 36 Months* |
| --- | --- | --- | --- | --- | --- | --- | --- |
| Loss of interest | -0,44 | -0,57 | -0,73 | -0,58 | -0,65 | -0,58 | -0,71 |
| Depressed mood | -0,43 | -0,47 | -0,63 | -0,61 | -0,65 | -0,60 | -0,72 |
| Change of sleep | -0,25 | -0,25 | -0,34 | -0,20 | -0,24 | -0,28 | -0,40 |
| Fatigue | -0,40 | -0,48 | -0,53 | -0,46 | -0,49 | -0,44 | -0,60 |
| Change of appetite | -0,20 | -0,24 | -0,26 | -0,22 | -0,34 | -0,35 | -0,42 |
| Worthlessness | -0,51 | -0,58 | -0,69 | -0,67 | -0,70 | -0,71 | -0,75 |
| Concentration problems | -0,26 | -0,31 | -0,36 | -0,30 | -0,35 | -0,40 | -0,47 |
| Psychomotor agitation | -0,28 | -0,34 | -0,40 | -0,31 | -0,28 | -0,33 | -0,46 |
| Suicidality | -0,04 | -0,08 | -0,11 | -0,07 | -0,12 | -0,02 | 0,01 |
| Overall health | 0,31 | 0,38 | 0,48 | 0,42 | 0,58 | 0,50 | 0,64 |
| Limited activities | -0,01 | 0,06 | -0,02 | 0,08 | -0,01 | 0,05 | -0,06 |
| Limited climbing stairs | 0,02 | 0,04 | -0,01 | 0,11 | 0,06 | 0,03 | -0,01 |
| Physical: accomplished less | -0,22 | -0,19 | -0,21 | -0,03 | -0,19 | -0,05 | -0,16 |
| Physical: limited activities | -0,05 | -0,04 | -0,03 | 0,11 | -0,08 | 0,04 | 0,02 |
| Emotional: accomplished less | -0,50 | -0,65 | -0,74 | -0,78 | -0,81 | -0,89 | -1,04 |
| Emotional: limited activities | -0,37 | -0,46 | -0,48 | -0,53 | -0,52 | -0,57 | -0,75 |
| Less careful | -0,12 | -0,11 | -0,19 | -0,14 | -0,22 | -0,20 | -0,21 |
| Pain | 0,39 | 0,45 | 0,53 | 0,51 | 0,58 | 0,56 | 0,72 |
| Calm, peaceful | 0,44 | 0,54 | 0,64 | 0,62 | 0,61 | 0,63 | 0,74 |
| Lot of energy | 0,51 | 0,61 | 0,71 | 0,77 | 0,79 | 0,71 | 0,96 |
| Downhearted, blue | -0,37 | -0,42 | -0,55 | -0,47 | -0,58 | -0,47 | -0,58 |

Appendix A1. Standardized mean differences (Cohen’s d) relative to baseline assessment for PHQ-9 and SF-12 items for the whole sample at all main assessments. *: CAU group had access to Deprexis at this point.

| Item | 3 Months | 6 Months | 12 Months | 18 Months* | 24 Months* | 30 Months* | 36 Months* |
| --- | --- | --- | --- | --- | --- | --- | --- |
| Loss of interest | -0,55 | -0,67 | -0,78 | -0,54 | -0,66 | -0,45 | -0,83 |
| Depressed mood | -0,55 | -0,55 | -0,66 | -0,53 | -0,54 | -0,55 | -0,75 |
| Change of sleep | -0,38 | -0,39 | -0,35 | -0,10 | -0,15 | -0,22 | -0,38 |
| Fatigue | -0,62 | -0,62 | -0,64 | -0,45 | -0,42 | -0,48 | -0,57 |
| Change of appetite | -0,28 | -0,34 | -0,27 | -0,21 | -0,27 | -0,36 | -0,44 |
| Worthlessness | -0,70 | -0,70 | -0,73 | -0,73 | -0,65 | -0,62 | -0,69 |
| Concentration problems | -0,42 | -0,47 | -0,41 | -0,37 | -0,44 | -0,42 | -0,57 |
| Psychomotor agitation | -0,38 | -0,47 | -0,41 | -0,09 | -0,15 | -0,14 | -0,35 |
| Suicidality | -0,14 | -0,24 | -0,24 | -0,06 | -0,14 | -0,01 | -0,02 |
| Overall health | 0,41 | 0,48 | 0,49 | 0,42 | 0,52 | 0,50 | 0,52 |
| Limited activities | -0,03 | 0,07 | -0,03 | 0,17 | 0,10 | 0,02 | -0,11 |
| Limited climbing stairs | 0,02 | 0,00 | -0,04 | 0,12 | -0,04 | -0,10 | -0,10 |
| Physical: accomplished less | -0,29 | -0,22 | -0,22 | 0,04 | -0,21 | -0,05 | -0,32 |
| Physical: limited activities | -0,04 | -0,06 | -0,03 | 0,23 | -0,02 | 0,06 | 0,02 |
| Emotional: accomplished less | -0,65 | -0,70 | -0,78 | -0,74 | -0,76 | -0,79 | -1,07 |
| Emotional: limited activities | -0,46 | -0,51 | -0,49 | -0,46 | -0,45 | -0,41 | -0,80 |
| Less careful | -0,14 | -0,13 | -0,22 | -0,02 | -0,15 | -0,07 | -0,17 |
| Pain | 0,48 | 0,57 | 0,55 | 0,54 | 0,57 | 0,56 | 0,67 |
| Calm, peaceful | 0,57 | 0,66 | 0,69 | 0,56 | 0,51 | 0,62 | 0,72 |
| Lot of energy | 0,65 | 0,73 | 0,77 | 0,79 | 0,76 | 0,55 | 0,97 |
| Downhearted, blue | -0,53 | -0,50 | -0,65 | -0,51 | -0,52 | -0,48 | -0,59 |

Appendix A2. Standardized mean differences (Cohen’s d) relative to baseline assessment for PHQ-9 and SF-12 items for the Deprexis group at all main assessments. *: CAU group had access to Deprexis at this point.

| Item | 3 Months | 6 Months | 12 Months | 18 Months* | 24 Months* | 30 Months* | 36 Months* |
| --- | --- | --- | --- | --- | --- | --- | --- |
| Loss of interest | -0,34 | -0,46 | -0,69 | -0,60 | -0,65 | -0,66 | -0,65 |
| Depressed mood | -0,32 | -0,39 | -0,59 | -0,67 | -0,72 | -0,64 | -0,71 |
| Change of sleep | -0,14 | -0,11 | -0,32 | -0,26 | -0,30 | -0,32 | -0,42 |
| Fatigue | -0,20 | -0,35 | -0,43 | -0,46 | -0,52 | -0,42 | -0,61 |
| Change of appetite | -0,13 | -0,14 | -0,26 | -0,22 | -0,38 | -0,34 | -0,40 |
| Worthlessness | -0,34 | -0,47 | -0,66 | -0,65 | -0,73 | -0,78 | -0,80 |
| Concentration problems | -0,10 | -0,15 | -0,30 | -0,24 | -0,29 | -0,38 | -0,40 |
| Psychomotor agitation | -0,20 | -0,24 | -0,38 | -0,44 | -0,37 | -0,47 | -0,54 |
| Suicidality | 0,07 | 0,06 | 0,00 | -0,07 | -0,10 | -0,01 | 0,03 |
| Overall health | 0,21 | 0,28 | 0,47 | 0,43 | 0,61 | 0,50 | 0,71 |
| Limited activities | 0,00 | 0,04 | 0,00 | 0,02 | -0,08 | 0,07 | -0,04 |
| Limited climbing stairs | 0,03 | 0,07 | 0,02 | 0,11 | 0,11 | 0,10 | 0,04 |
| Physical: accomplished less | -0,16 | -0,16 | -0,20 | -0,06 | -0,18 | -0,05 | -0,07 |
| Physical: limited activities | -0,06 | -0,03 | -0,02 | 0,03 | -0,13 | 0,01 | 0,00 |
| Emotional: accomplished less | -0,35 | -0,59 | -0,70 | -0,79 | -0,84 | -0,94 | -1,01 |
| Emotional: limited activities | -0,29 | -0,42 | -0,47 | -0,56 | -0,55 | -0,66 | -0,72 |
| Less careful | -0,11 | -0,10 | -0,17 | -0,21 | -0,26 | -0,29 | -0,24 |
| Pain | 0,30 | 0,34 | 0,52 | 0,49 | 0,58 | 0,55 | 0,74 |
| Calm, peaceful | 0,30 | 0,43 | 0,59 | 0,65 | 0,66 | 0,64 | 0,76 |
| Lot of energy | 0,37 | 0,50 | 0,65 | 0,75 | 0,80 | 0,81 | 0,95 |
| Downhearted, blue | -0,21 | -0,35 | -0,46 | -0,43 | -0,59 | -0,46 | -0,57 |

Appendix A3. Standardized mean differences (Cohen’s d) relative to baseline assessment for PHQ-9 and SF-12 items for the CAU group at all main assessment. *: CAU group had access to Deprexis at this point.

| Item | 3 Months | 6 Months | 12 Months | 18 Months* | 24 Months* | 30 Months* | 36 Months* |
| --- | --- | --- | --- | --- | --- | --- | --- |
| Loss of interest | -0,20 | -0,22 | -0,09 | 0,06 | 0,00 | 0,17 | -0,13 |
| Depressed mood | -0,27 | -0,20 | -0,13 | 0,09 | 0,12 | 0,04 | -0,04 |
| Change of sleep | -0,25 | -0,30 | -0,06 | 0,13 | 0,11 | 0,07 | 0,00 |
| Fatigue | -0,35 | -0,21 | -0,16 | 0,06 | 0,13 | -0,02 | 0,09 |
| Change of appetite | -0,14 | -0,19 | 0,00 | 0,02 | 0,10 | -0,01 | -0,04 |
| Worthlessness | -0,39 | -0,24 | -0,11 | -0,12 | 0,03 | 0,10 | 0,07 |
| Concentration problems | -0,25 | -0,25 | -0,05 | -0,05 | -0,07 | 0,02 | -0,09 |
| Psychomotor agitation | -0,22 | -0,26 | -0,09 | 0,29 | 0,16 | 0,27 | 0,17 |
| Suicidality | -0,16 | -0,24 | -0,18 | 0,04 | 0,00 | 0,03 | -0,02 |
| Overall health | 0,21 | 0,21 | 0,03 | 0,00 | -0,09 | 0,02 | -0,15 |
| Limited activities | -0,05 | 0,00 | -0,06 | 0,11 | 0,14 | -0,08 | -0,08 |
| Limited climbing stairs | 0,00 | -0,05 | -0,05 | 0,02 | -0,13 | -0,18 | -0,13 |
| Physical: accomplished less | -0,14 | -0,07 | -0,03 | 0,09 | -0,04 | -0,01 | -0,26 |
| Physical: limited activities | -0,07 | -0,11 | -0,09 | 0,11 | 0,03 | -0,04 | -0,07 |
| Emotional: accomplished less | -0,29 | -0,12 | -0,09 | 0,06 | 0,09 | 0,14 | -0,02 |
| Emotional: limited activities | -0,17 | -0,08 | -0,01 | 0,10 | 0,10 | 0,25 | -0,07 |
| Less careful | -0,05 | -0,04 | -0,06 | 0,19 | 0,10 | 0,20 | 0,05 |
| Pain | 0,14 | 0,18 | 0,01 | 0,00 | -0,06 | -0,04 | -0,11 |
| Calm, peaceful | 0,29 | 0,23 | 0,12 | -0,08 | -0,13 | -0,01 | -0,04 |
| Lot of energy | 0,26 | 0,20 | 0,10 | -0,03 | -0,05 | -0,21 | -0,03 |
| Downhearted, blue | -0,27 | -0,10 | -0,14 | -0,02 | 0,12 | 0,03 | 0,02 |

Appendix A4. Item-level between-group (Deprexis vs. CAU) standardized mean differences (Cohen’s d) at all points of assessment. Values below zero indicate lower scores for the Deprexis group. *: CAU group had access to Deprexis at this point.

|  | Baseline | 3 Months | 4 Months | 5 Months | 6 Months | 7 Months | 8 Months | 9 Months | 10 Months | 11 Months | 12 Months |
| --- | --- | --- | --- | --- | --- | --- | --- | --- | --- | --- | --- |
| Treatment | -0.04  -0.04 | -0.36  -0.62 | -0.34  -0.56 | -0.32  -0.39 | -0.23  -0.33 | -0.24  -0.39 | -0.18  -0.25 | -0.07  -0.13 | -0.14  -0.23 | -0.09  -0.10 | -0.13  -0.17 |
| Loss of interest | 0  0 | 0  -0.01 | -0.02  -0.02 | -0.02  -0.03 | 0  -0.01 | -0.02  -0.03 | 0  0.01 | 0  -0.01 | 0  0 | 0  0 | 0  0 |
| Depressed mood | 0  0 | 0  -0.03 | -0.01  -0.04 | -0.01  0 | 0  -0.01 | 0.04  0 | 0.03  -0.03 | 0  -0.01 | 0  -0.03 | 0.09  0.05 | 0  0 |
| Change of sleep | -0.02  -0.02 | -0.05  -0.06 | -0.17  -0.18 | -0.17  -0.10 | -0.12  -0.12 | -0.09  -0.10 | 0  0.01 | -0.02  -0.03 | 0  0 | 0  -0.02 | 0  -0.01 |
| Fatigue | 0  0 | -0.06  -0.08 | -0.03  -0.08 | -0.03  -0.07 | 0  -0.03 | -0.04  -0.07 | -0.02  -0.01 | -0.03  -0.04 | 0  -0.01 | -0.05  -0.04 | -0.03  -0.03 |
| Change of appetite | 0  0 | 0  -0.03 | 0.03  -0.01 | 0.03  -0.02 | 0  -0.02 | 0  -0.02 | 0.07  0.04 | 0  -0.01 | 0  -0.01 | 0  0 | 0  0 |
| Worthlessness | -0.02  -0.02 | -0.12  -0.13 | -0.04  -0.04 | -0.04  -0.09 | -0.01  -0.02 | -0.09  -0.09 | -0.17  -0.16 | -0.02  -0.02 | -0.09  -0.10 | -0.05  -0.04 | 0  -0.01 |
| Concentration problems | 0  0 | 0  -0.01 | -0.03 | 0  -0.01 | -0.04  -0.05 | 0.01  -0.01 | 0  -0.03 | 0  -0.01 | 0  -0.01 | 0  -0.01 | 0  -0.01 |
| Psychomotor agitation | -0.03  -0.03 | -0.03  -0.04 | -0.07  -0.07 | -0.07  -0.05 | -0.05  -0.06 | -0.05  -0.05 | -0.06  -0.04 | 0  0 | 0  -0.01 | 0  -0.01 | 0  0 |
| Suicidality | 0  0 | 0  -0.02 | 0  -0.02 | 0  -0.01 | -0.04  -0.05 | 0  -0.01 | -0.04  -0.05 | 0  0 | -0.06  -0.07 | -0.07  -0.06 | -0.03  -0.05 |
| Overall health | 0  0 | 0  0.01 | - | - | 0  0 | - | - | - | - | - | -0.03  -0.03 |
| Limited activities | 0  0 | 0  0 | - | - | 0.02  0.02 | - | - | - | - | - | 0  0 |
| Limited climbing stairs | 0  0 | 0  0 | - | - | 0  0.01 | - | - | - | - | - | 0  0 |
| Physical: accomplished less | 0  0 | 0  -0.03 | - | - | 0  0.01 | - | - | - | - | - | 0  0 |
| Physical: limited activities | 0  0 | 0  0 | - | - | 0  0 | - | - | - | - | - | 0  0 |
| Emotional: accomplished less | 0  0 | -0.09  -0.10 | - | - | 0  0 | - | - | - | - | - | 0  0 |
| Emotional: limited activities | 0  0 | 0  -0.09 | - | - | 0  -0.01 | - | - | - | - | - | 0  0 |
| Less careful | 0  0 | 0  0 | - | - | 0  0 | - | - | - | - | - | 0  0 |
| Pain | 0  0 | -0.02  -0.01 | - | - | 0  0.01 | - | - | - | - | - | -0.02  -0.03 |
| Calm, peaceful | 0  -0.01 | 0.02  0.04 | - | - | 0  0 | - | - | - | - | - | 0 |
| Lot of energy | 0  0 | 0  0.02 | - | - | 0  0 | - | - | - | - | - | 0 |
| Downhearted, blue | 0.03  0.02 | -0.02  -0.04 | - | - | 0  0 | - | - | - | - | - | 0 |

Appendix A5. One-step (first value) and two-step (second value) Bridge Expected Influence (BEI) values for all network models.

|  | Baseline | 3 Months | 4 Months | 5 Months | 6 Months | 7 Months | 8 Months | 9 Months | 10 Months | 11 Months | 12 Months |
| --- | --- | --- | --- | --- | --- | --- | --- | --- | --- | --- | --- |
| Treatment | -0.04  -0.04 | -0.36  -0.62 | -0.34  -0.56 | -0.32  -0.39 | -0.23  -0.33 | -0.24  -0.39 | -0.18  -0.25 | -0.07  -0.13 | -0.14  -0.23 | -0.09  -0.10 | -0.13  -0.17 |
| Loss of interest | 0  0 | 0  -0.01 | -0.02  -0.02 | -0.02  -0.03 | 0  -0.01 | -0.02  -0.03 | 0  0.01 | 0  -0.01 | 0  0 | 0  0 | 0  0 |
| Depressed mood | 0  0 | 0  -0.03 | -0.01  -0.04 | -0.01  0 | 0  -0.01 | 0.04  0 | 0.03  -0.03 | 0  -0.01 | 0  -0.03 | 0.09  0.05 | 0  0 |
| Change of sleep | -0.02  -0.02 | -0.05  -0.06 | -0.17  -0.18 | -0.17  -0.10 | -0.12  -0.12 | -0.09  -0.10 | 0  0.01 | -0.02  -0.03 | 0  0 | 0  -0.02 | 0  -0.01 |
| Fatigue | 0  0 | -0.06  -0.08 | -0.03  -0.08 | -0.03  -0.07 | 0  -0.03 | -0.04  -0.07 | -0.02  -0.01 | -0.03  -0.04 | 0  -0.01 | -0.05  -0.04 | -0.03  -0.03 |
| Change of appetite | 0  0 | 0  -0.03 | 0.03  -0.01 | 0.03  -0.02 | 0  -0.02 | 0  -0.02 | 0.07  0.04 | 0  -0.01 | 0  -0.01 | 0  0 | 0  0 |
| Worthlessness | -0.02  -0.02 | -0.12  -0.13 | -0.04  -0.04 | -0.04  -0.09 | -0.01  -0.02 | -0.09  -0.09 | -0.17  -0.16 | -0.02  -0.02 | -0.09  -0.10 | -0.05  -0.04 | 0  -0.01 |
| Concentration problems | 0  0 | 0  -0.01 | -0.03 | 0  -0.01 | -0.04  -0.05 | 0.01  -0.01 | 0  -0.03 | 0  -0.01 | 0  -0.01 | 0  -0.01 | 0  -0.01 |
| Psychomotor agitation | -0.03  -0.03 | -0.03  -0.04 | -0.07  -0.07 | -0.07  -0.05 | -0.05  -0.06 | -0.05  -0.05 | -0.06  -0.04 | 0  0 | 0  -0.01 | 0  -0.01 | 0  0 |
| Suicidality | 0  0 | 0  -0.02 | 0  -0.02 | 0  -0.01 | -0.04  -0.05 | 0  -0.01 | -0.04  -0.05 | 0  0 | -0.06  -0.07 | -0.07  -0.06 | -0.03  -0.05 |
| Overall health | 0  0 | 0  0.01 | - | - | 0  0 | - | - | - | - | - | -0.03  -0.03 |
| Limited activities | 0  0 | 0  0 | - | - | 0.02  0.02 | - | - | - | - | - | 0  0 |
| Limited climbing stairs | 0  0 | 0  0 | - | - | 0  0.01 | - | - | - | - | - | 0  0 |
| Physical: accomplished less | 0  0 | 0  -0.03 | - | - | 0  0.01 | - | - | - | - | - | 0  0 |
| Physical: limited activities | 0  0 | 0  0 | - | - | 0  0 | - | - | - | - | - | 0  0 |
| Emotional: accomplished less | 0  0 | -0.09  -0.10 | - | - | 0  0 | - | - | - | - | - | 0  0 |
| Emotional: limited activities | 0  0 | 0  -0.09 | - | - | 0  -0.01 | - | - | - | - | - | 0  0 |
| Less careful | 0  0 | 0  0 | - | - | 0  0 | - | - | - | - | - | 0  0 |
| Pain | 0  0 | -0.02  -0.01 | - | - | 0  0.01 | - | - | - | - | - | -0.02  -0.03 |
| Calm, peaceful | 0  -0.01 | 0.02  0.04 | - | - | 0  0 | - | - | - | - | - | 0 |
| Lot of energy | 0  0 | 0  0.02 | - | - | 0  0 | - | - | - | - | - | 0 |
| Downhearted, blue | 0.03  0.02 | -0.02  -0.04 | - | - | 0  0 | - | - | - | - | - | 0 |

Appendix A5. One-step (first value) and two-step (second value) Bridge Expected Influence (BEI) values for all network models.
