# Supplementary figures and images for: Maintaining Outcomes of Internet-Delivered Cognitive-Behavioral Therapy for Depression: A Network Analysis of Follow-Up Effects
Source: Front Psychiatry. 2021 Apr 20;12:598317. doi: 10.3389/fpsyt.2021.598317 (PMC8095668; doi:10.3389/fpsyt.2021.598317)

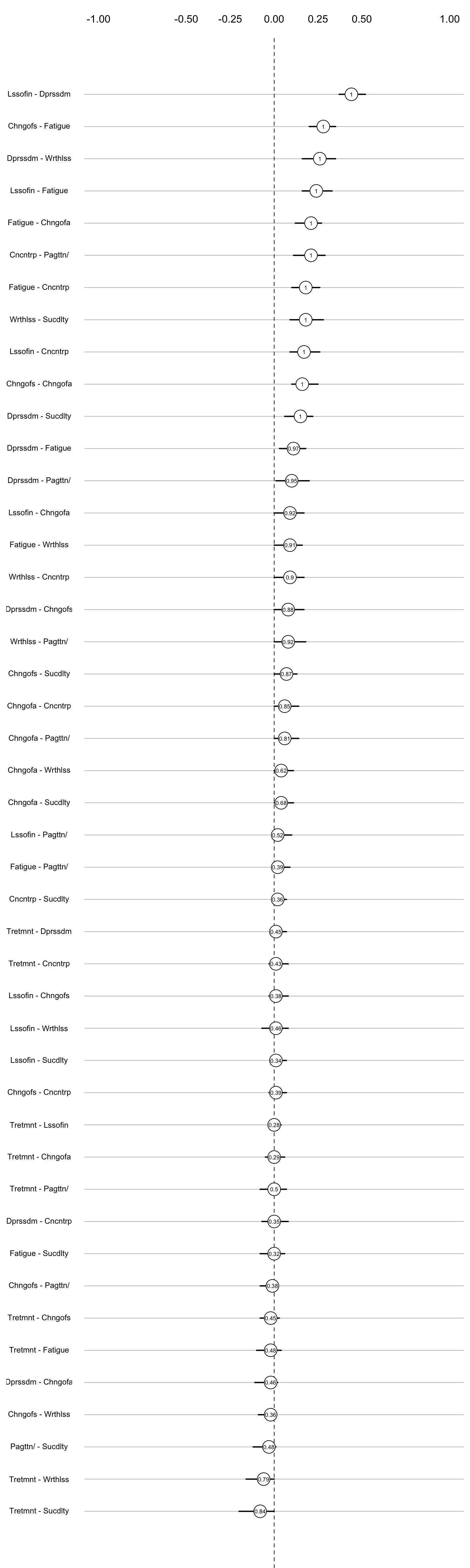

Supplement: Supplementary file 2 [file Data_Sheet_1.ZIP › Network-Graphs/J-11Months.png]

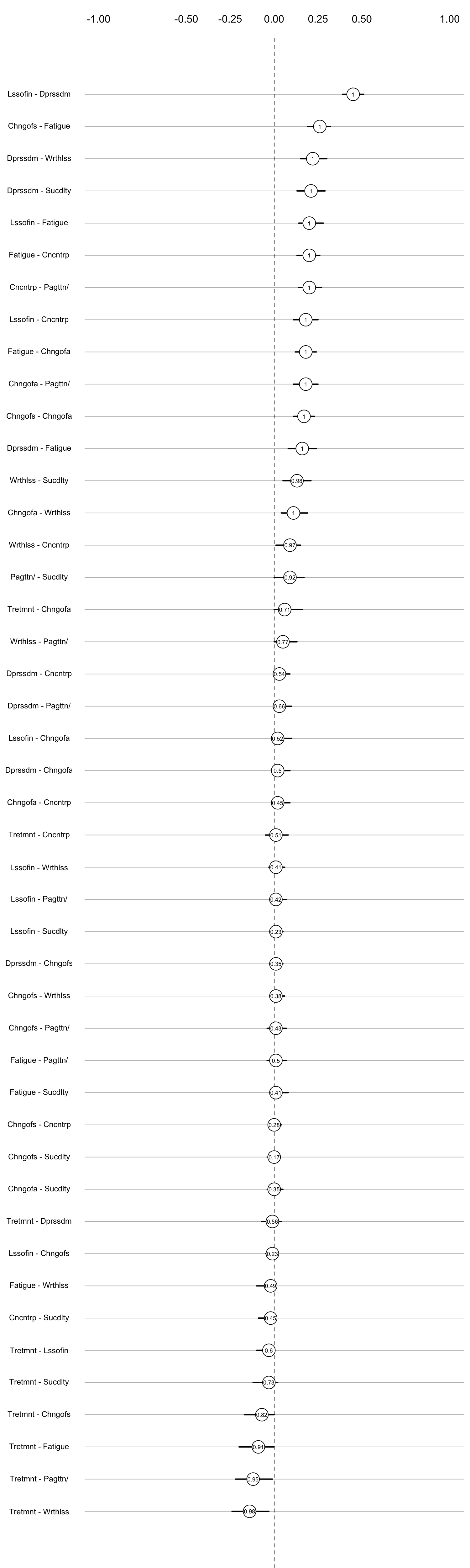

Supplement: Supplementary file 2 [file Data_Sheet_1.ZIP › Network-Graphs/C-4Months.png]

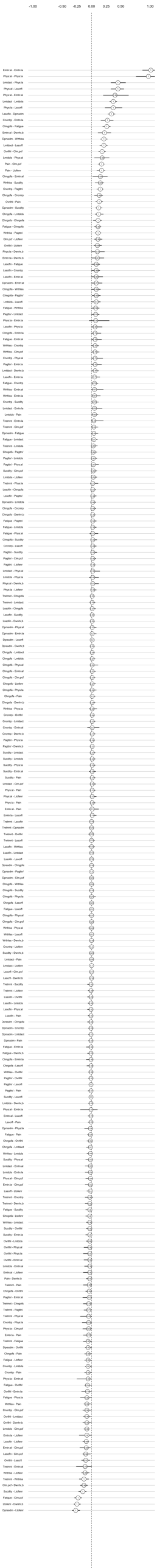

Supplement: Supplementary file 2 [file Data_Sheet_1.ZIP › Network-Graphs/B-Post-Bootstrap.png]

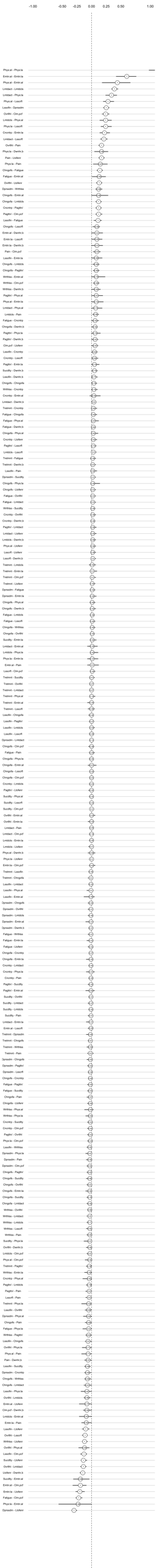

Supplement: Supplementary file 2 [file Data_Sheet_1.ZIP › Network-Graphs/A-Pre-Bootstrap.png]

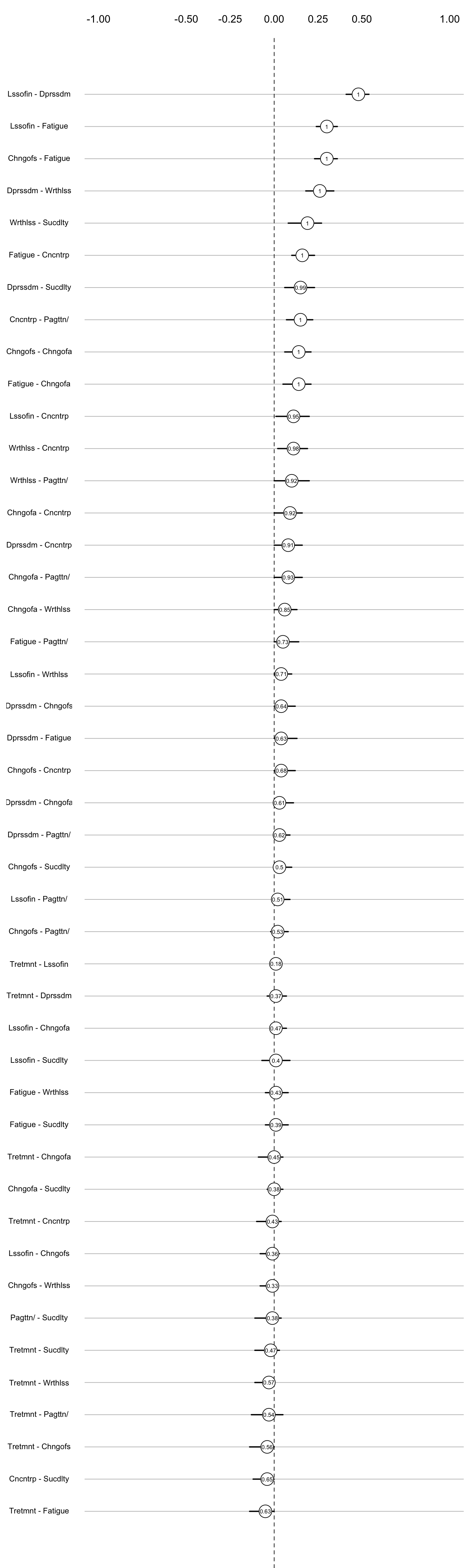

Supplement: Supplementary file 2 [file Data_Sheet_1.ZIP › Network-Graphs/I-10Months.png]

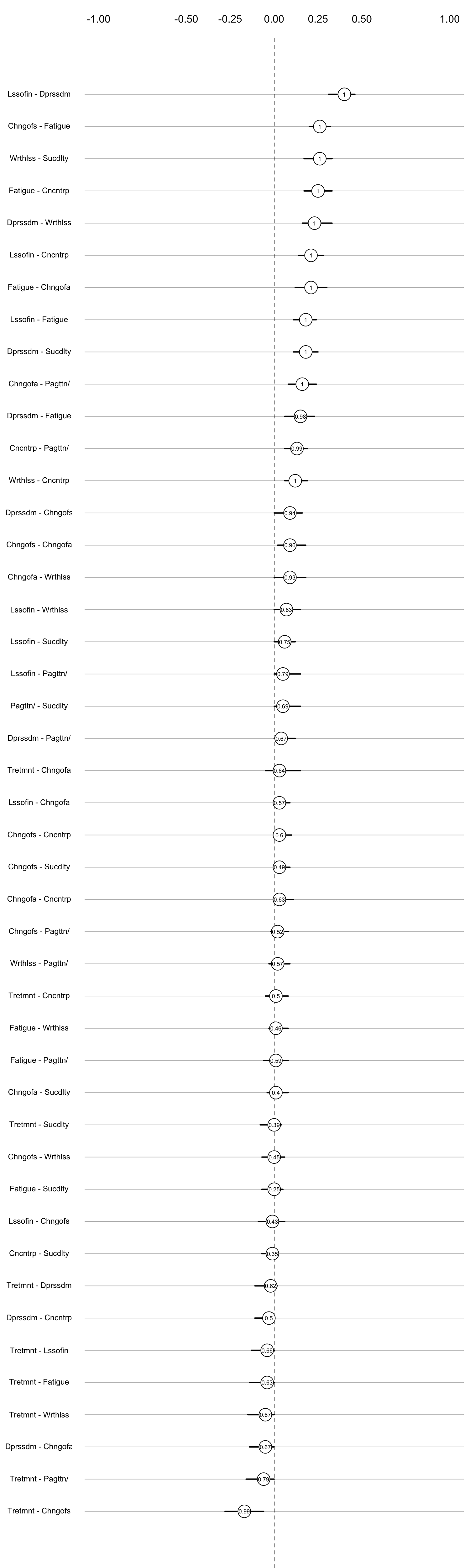

Supplement: Supplementary file 2 [file Data_Sheet_1.ZIP › Network-Graphs/D-5Months.png]

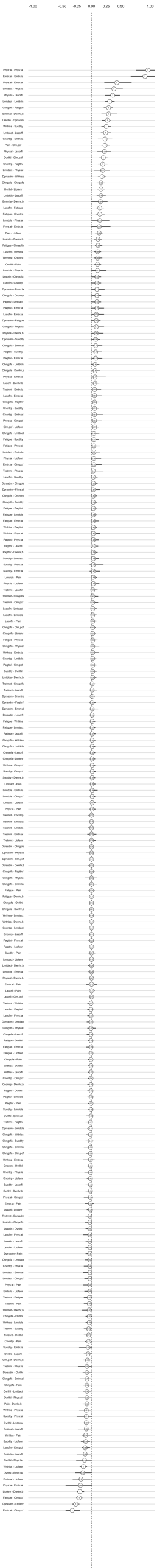

Supplement: Supplementary file 2 [file Data_Sheet_1.ZIP › Network-Graphs/G-8Months.png]

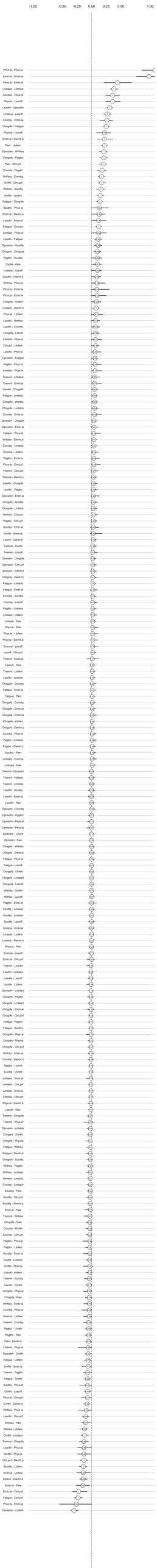

Supplement: Supplementary file 2 [file Data_Sheet_1.ZIP › Network-Graphs/E-6Months.png]

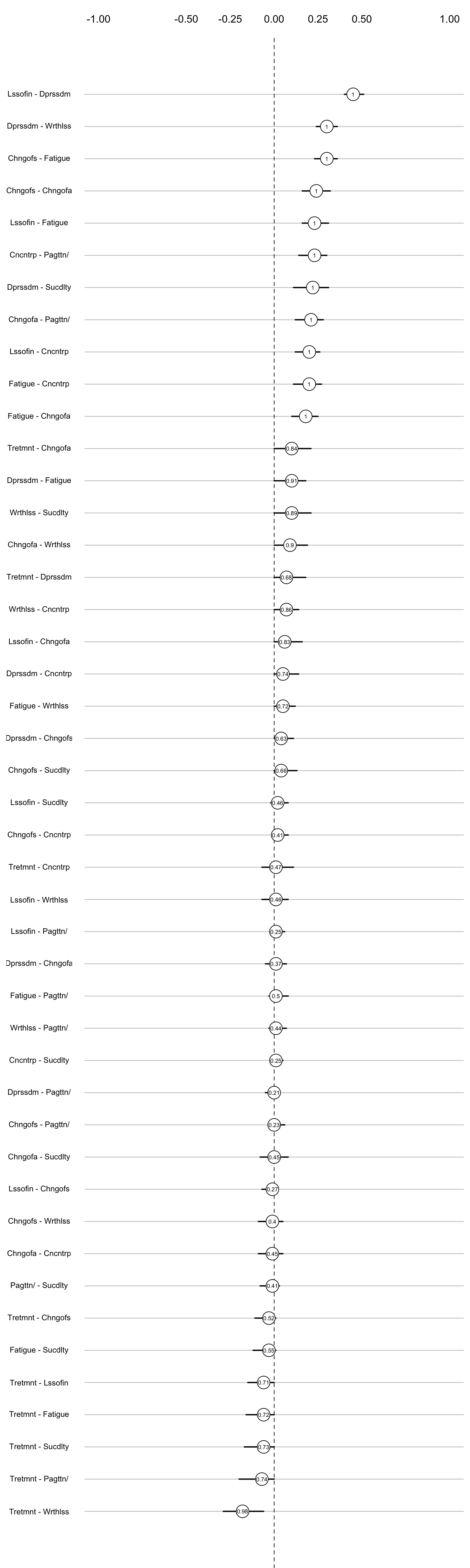

Supplement: Supplementary file 2 [file Data_Sheet_1.ZIP › Network-Graphs/H-9Months.png]

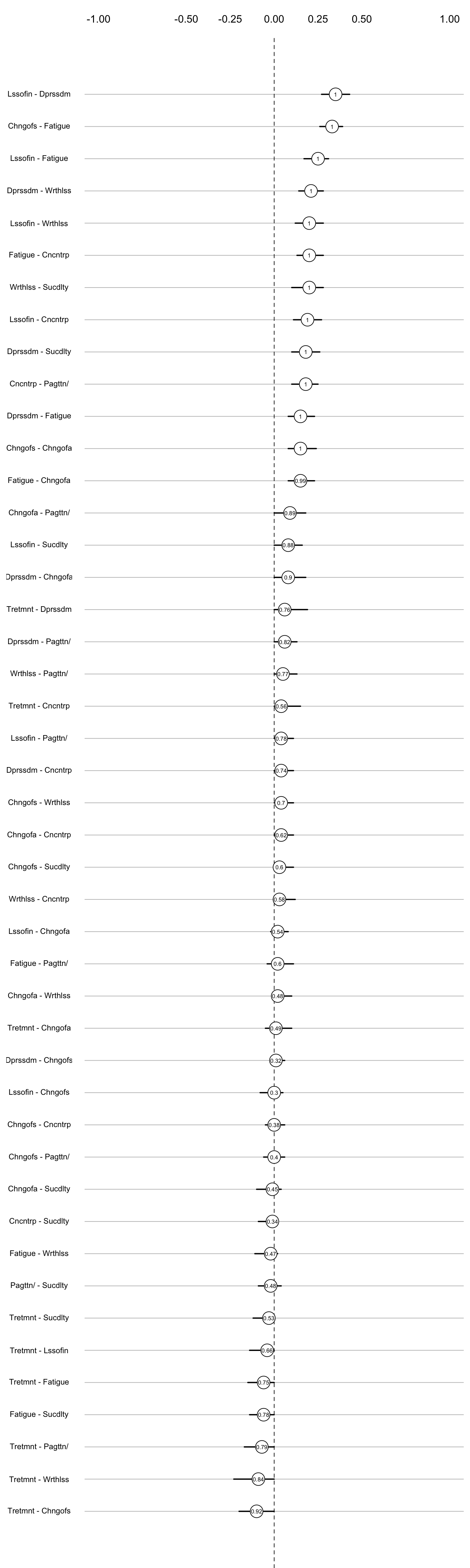

Supplement: Supplementary file 2 [file Data_Sheet_1.ZIP › Network-Graphs/F-7Months.png]

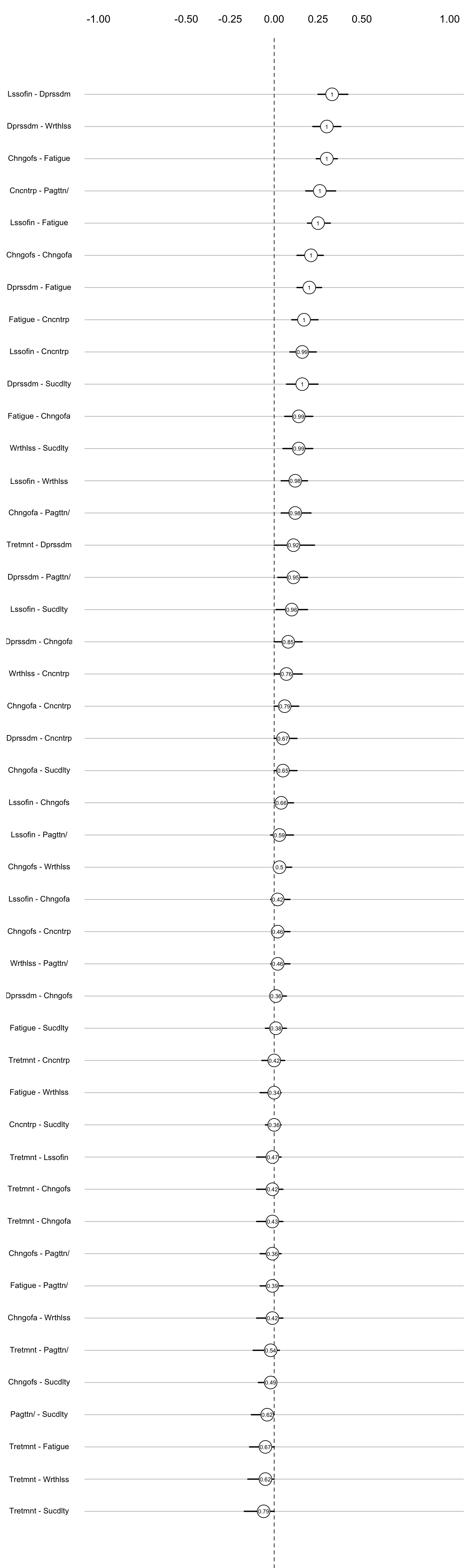

Supplement: Supplementary file 2 [file Data_Sheet_1.ZIP › Network-Graphs/K-12Months.png]
