# Supplementary material for: Maintaining Outcomes of Internet-Delivered Cognitive-Behavioral Therapy for Depression: A Network Analysis of Follow-Up Effects
Source: Front Psychiatry. 2021 Apr 20;12:598317. doi: 10.3389/fpsyt.2021.598317 (PMC8095668; doi:10.3389/fpsyt.2021.598317)
Supplement: Supplementary file 3 [file Data_Sheet_2.docx]

This supplementary file contains covariance matrices used for all network analyses in the paper “Maintaining Outcomes of Internet-delivered Cognitive-Behavioral Therapy for Depression: A Network Intervention Analysis of Follow-up Effects”.

Instructions:

- The matrices are provided in raw csv format. They are separated by headlines (e.g. “Baseline”, “4 months”, etc.).
- Copy all text below but not including that headline up to the next headline.
- Paste the text to a text editor.
- Save the text file as CSV file.
- You can now open the matrix using any statistical or spreadsheet software.

**Baseline**:

"","Treatment","PHQ1.1","PHQ1.2","PHQ1.3","PHQ1.4","PHQ1.5","PHQ1.6","PHQ1.7","PHQ1.8","PHQ1.9","SF1.1","SF1.2","SF1.3","SF1.4","SF1.5","SF1.6","SF1.7","SF1.8","SF1.9","SF1.10","SF1.11","SF1.12"

"Treatment",0.250250733780083,-0.00389609592153363,-0.0107191249493415,-0.00520707540025299,0.00717814993266007,0.000110527543873294,-0.00970390899080165,0.0124189976379855,-0.0118765939504591,0.00190045971271026,0.00411203399336016,-0.0044313357867719,0.00158320472566654,-0.00171215352685205,-0.00964250479976093,0.00032953582525186,0.00103466061903612,-0.000166814718993954,-0.00603910218885473,0.0032104157882456,0.00609027234805533,0.0102749679674803

"PHQ1.1",-0.00389609592153363,0.426024324246878,0.151702124175649,-0.0340557877543669,0.101907418854362,0.00786280666276409,0.0303787001142118,0.0372355014470921,-0.0101767212618152,-0.00810637662055894,-0.0849486046920989,0.00825272327587266,0.0107662014958061,0.00182165766754134,-0.0155229794950938,0.0170887863666321,0.0472044718625529,0.0102596169197202,-0.0805090816798549,-0.162106040944315,-0.165906960369735,0.0977964082641854

"PHQ1.2",-0.0107191249493415,0.151702124175649,0.378835203432085,-0.0374227842297663,0.0470018380321185,-0.012809937654278,0.110063942230937,-0.0228689675499318,-0.0261807002533946,0.029374741590696,-0.0630406127319543,-0.0130883033203293,-0.0177703728871841,-0.0237961708346467,-0.0254827392818985,0.0115337538838151,0.0333967161038631,-0.041835698759226,-0.114378610054732,-0.0971056111149773,-0.23288869876332,0.0756069804284375

"PHQ1.3",-0.00520707540025299,-0.0340557877543669,-0.0374227842297663,0.757455492195527,0.112756516008073,0.0697776758923052,-0.0760265757338824,-0.00456335479750945,-0.0101091766516704,-0.027325888416304,-0.0521976559973473,0.0244818509679347,0.0707069259833881,0.0506523171894892,0.0355008330501918,0.0238678090575275,-0.00138057089523217,0.152803306001646,-0.0612813826586377,-0.0532701825341919,0.0207863420704675,0.0424824895715216

"PHQ1.4",0.00717814993266007,0.101907418854362,0.0470018380321185,0.112756516008073,0.544366574833288,0.0471492080906162,-0.0141884617431422,0.0540633200018012,-0.0198826770589849,-0.0164716742466729,-0.0668937257197595,0.0460695177314836,0.049778330870343,0.0404571746703618,0.0109933970026568,0.0341304961867997,0.0256301093403962,0.0871560853600128,-0.0718787226290818,-0.209015772689872,-0.0812254639086633,0.0963554565810965

"PHQ1.5",0.000110527543873294,0.00786280666276409,-0.012809937654278,0.0697776758923052,0.0471492080906162,0.727488200161288,-0.00182370447390936,0.00409054252649591,0.0538821776382311,-0.0183475722829669,-0.0313857288472796,0.0274814457002739,0.0808887642611234,0.0457461223253358,0.023568975327796,0.0094859241126071,0.0110650352255376,0.0697479971999689,-0.0552647953398313,-0.0326424679572463,-0.041320926957668,0.0867610517309841

"PHQ1.6",-0.00970390899080165,0.0303787001142118,0.110063942230937,-0.0760265757338824,-0.0141884617431422,-0.00182370447390936,0.661126644097215,0.024337551118989,-0.025190045971271,0.0308893783030338,-0.0162291276920621,-0.0544133238907333,-0.0549690318196518,-0.0400693048636213,-0.0372549461075883,0.0127812823651257,0.0117742536320579,-0.0959317676629156,-0.0840592673251925,0.0254694350405063,-0.154639291313763,0.0814751742855622

"PHQ1.7",0.0124189976379855,0.0372355014470921,-0.0228689675499318,-0.00456335479750945,0.0540633200018012,0.00409054252649591,0.024337551118989,0.530327529955011,0.06488887888228,-0.0170191949501193,-0.00483046302853657,0.00122296680489432,-0.00501262879529071,0.00513236696782011,0.00490517146096945,0.0179126259297618,0.0583943622765399,0.0690531064380247,-0.0353841650872144,-0.0351528759676277,-0.00138978152388828,0.0465607512598093

"PHQ1.8",-0.0118765939504591,-0.0101767212618152,-0.0261807002533946,-0.0101091766516704,-0.0198826770589849,0.0538821776382311,-0.025190045971271,0.06488887888228,0.399937777086412,-0.00621819774605683,-0.0216992177106061,0.033043642005379,0.00409975315515202,0.0383008641616486,0.0340967238817273,0.00275397796817625,0.0241338938853707,0.0861879459479374,-0.0427465275929967,0.0544501664053577,-0.000121784978897426,0.0525691513531437

"PHQ1.9",0.00190045971271026,-0.00810637662055894,0.029374741590696,-0.027325888416304,-0.0164716742466729,-0.0183475722829669,0.0308893783030338,-0.0170191949501193,-0.00621819774605683,0.200718019673903,-0.0125571570678271,-0.0123739678978889,-0.0128119844606461,-0.00930478174903698,-0.00966808987936123,-0.0121928255343188,0.00537184331287891,-0.0208917525984207,-0.0268612633707626,-0.000540356881158329,-0.0696896632184802,0.0311974226614214

"SF1.1",0.00411203399336016,-0.0849486046920989,-0.0630406127319543,-0.0521976559973473,-0.0668937257197595,-0.0313857288472796,-0.0162291276920621,-0.00483046302853657,-0.0216992177106061,-0.0125571570678271,0.422902944535641,-0.135426943340306,-0.109865402013239,-0.0981198036703332,-0.072914406651302,-0.023138122587327,-0.0433554524874838,-0.231683129812553,0.196100424507641,0.224513167105366,0.165062652742925,-0.148292144766521

"SF1.2",-0.0044313357867719,0.00825272327587266,-0.0130883033203293,0.0244818509679347,0.0460695177314836,0.0274814457002739,-0.0544133238907333,0.00122296680489432,0.033043642005379,-0.0123739678978889,-0.135426943340306,0.336063090759488,0.179942935038459,0.114412382359804,0.120098410450175,0.00776763016665097,0.0157757600815448,0.284799801869144,-0.0345347404444845,-0.0806175624173602,-0.00947876029031901,0.0946801455688689

"SF1.3",0.00158320472566654,0.0107662014958061,-0.0177703728871841,0.0707069259833881,0.049778330870343,0.0808887642611234,-0.0549690318196518,-0.00501262879529071,0.00409975315515202,-0.0128119844606461,-0.109865402013239,0.179942935038459,0.332061584310001,0.104776017979147,0.0856731741463794,0.00329535825251859,0.0106106442118363,0.200276932901594,0.000733780082936594,-0.0856567996954352,-0.0103875423177217,0.0783261626883574

"SF1.4",-0.00171215352685205,0.00182165766754134,-0.0237961708346467,0.0506523171894892,0.0404571746703618,0.0457461223253358,-0.0400693048636213,0.00513236696782011,0.0383008641616486,-0.00930478174903698,-0.0981198036703332,0.114412382359804,0.104776017979147,0.248736097067745,0.119285828322069,0.0232558139534884,0.0301238727213928,0.228640552146486,-0.0494498184482752,-0.0746408878227302,-0.00989323857984387,0.0729829746646308

"SF1.5",-0.00964250479976093,-0.0155229794950938,-0.0254827392818985,0.0355008330501918,0.0109933970026568,0.023568975327796,-0.0372549461075883,0.00490517146096945,0.0340967238817273,-0.00966808987936123,-0.072914406651302,0.120098410450175,0.0856731741463794,0.119285828322069,0.216167314139748,0.00331889652575087,0.0145476762607304,0.203725801631714,0.00208467228583242,-0.0442847025785667,0.00245412083526074,0.0733329785535629

"SF1.6",0.00032953582525186,0.0170887863666321,0.0115337538838151,0.0238678090575275,0.0341304961867997,0.0094859241126071,0.0127812823651257,0.0179126259297618,0.00275397796817625,-0.0121928255343188,-0.023138122587327,0.00776763016665097,0.00329535825251859,0.0232558139534884,0.00331889652575087,0.102862254025045,0.0368138593352792,0.0249976461726768,-0.0318237454100367,-0.0500894454382826,-0.0347660295640712,0.0390970718388099

"SF1.7",0.00103466061903612,0.0472044718625529,0.0333967161038631,-0.00138057089523217,0.0256301093403962,0.0110650352255376,0.0117742536320579,0.0583943622765399,0.0241338938853707,0.00537184331287891,-0.0433554524874838,0.0157757600815448,0.0106106442118363,0.0301238727213928,0.0145476762607304,0.0368138593352792,0.217278729997585,0.0692199211570187,-0.0799523503477524,-0.0535454779906911,-0.0904760052889477,0.0747473217538674

"SF1.8",-0.000166814718993954,0.0102596169197202,-0.041835698759226,0.152803306001646,0.0871560853600128,0.0697479971999689,-0.0959317676629156,0.0690531064380247,0.0861879459479374,-0.0208917525984207,-0.231683129812553,0.284799801869144,0.200276932901594,0.228640552146486,0.203725801631714,0.0249976461726768,0.0692199211570187,1.24252608654716,-0.135542587900099,-0.12651003139801,-0.0214566711559953,0.1636176074471

"SF1.9",-0.00603910218885473,-0.0805090816798549,-0.114378610054732,-0.0612813826586377,-0.0718787226290818,-0.0552647953398313,-0.0840592673251925,-0.0353841650872144,-0.0427465275929967,-0.0268612633707626,0.196100424507641,-0.0345347404444845,0.000733780082936594,-0.0494498184482752,0.00208467228583242,-0.0318237454100367,-0.0799523503477524,-0.135542587900099,0.963067425895375,0.201564374107081,0.274711093281153,-0.187474159069604

"SF1.10",0.0032104157882456,-0.162106040944315,-0.0971056111149773,-0.0532701825341919,-0.209015772689872,-0.0326424679572463,0.0254694350405063,-0.0351528759676277,0.0544501664053577,-0.000540356881158329,0.224513167105366,-0.0806175624173602,-0.0856567996954352,-0.0746408878227302,-0.0442847025785667,-0.0500894454382826,-0.0535454779906911,-0.12651003139801,0.201564374107081,0.733368674856621,0.193579782465419,-0.181976437165091

"SF1.11",0.00609027234805533,-0.165906960369735,-0.23288869876332,0.0207863420704675,-0.0812254639086633,-0.041320926957668,-0.154639291313763,-0.00138978152388828,-0.000121784978897426,-0.0696896632184802,0.165062652742925,-0.00947876029031901,-0.0103875423177217,-0.00989323857984387,0.00245412083526074,-0.0347660295640712,-0.0904760052889477,-0.0214566711559953,0.274711093281153,0.193579782465419,0.681604941809295,-0.225181449384525

"SF1.12",0.0102749679674803,0.0977964082641854,0.0756069804284375,0.0424824895715216,0.0963554565810965,0.0867610517309841,0.0814751742855622,0.0465607512598093,0.0525691513531437,0.0311974226614214,-0.148292144766521,0.0946801455688689,0.0783261626883574,0.0729829746646308,0.0733329785535629,0.0390970718388099,0.0747473217538674,0.1636176074471,-0.187474159069604,-0.181976437165091,-0.225181449384525,0.761021028888625

**3 Months**

"","Treatment","PHQ2.1","PHQ2.2","PHQ2.3","PHQ2.4","PHQ2.5","PHQ2.6","PHQ2.7","PHQ2.8","PHQ2.9","SF2.1","SF2.2","SF2.3","SF2.4","SF2.5","SF2.6","SF2.7","SF2.8","SF2.9","SF2.10","SF2.11","SF2.12"

"Treatment",0.250302367002088,-0.0382794881893881,-0.0456234337040742,-0.0649330958546965,-0.0697988518767667,-0.0375597327319686,-0.0740616166439528,-0.0516446325064569,-0.0365524236414398,-0.0218697608599422,0.0388040557261513,-0.00976985252855495,-0.00189959672781393,-0.0202124062836568,-0.0077134083644995,-0.0333631924378623,-0.0204476774380191,-0.0226783965312318,0.037244295110194,0.0779793170514076,0.0664231464989909,-0.0666514466561869

"PHQ2.1",-0.0382794881893881,0.504715879582995,0.322211688270949,0.199069023328442,0.274582350132275,0.19248317375559,0.228500573364517,0.221071233134544,0.105795338494195,0.111785167809329,-0.23748096046399,0.0915396492891326,0.0651788235048082,0.0739030264584197,0.067723237470504,0.122964904514766,0.134712777489256,0.184639059194222,-0.310336594598174,-0.387550844710582,-0.43888701059243,0.2993921290471

"PHQ2.2",-0.0456234337040742,0.322211688270949,0.497933099340195,0.192014374196157,0.272006566679331,0.18261921276529,0.297241576421299,0.195289000115021,0.103470510939237,0.146173096830636,-0.258711132333925,0.0895738280882389,0.07494519053478,0.0765833748688581,0.0555239924294971,0.127875972018417,0.132809695262859,0.202882158778402,-0.331619048946857,-0.368324834525955,-0.506282611196119,0.322758911548502

"PHQ2.3",-0.0649330958546965,0.199069023328442,0.192014374196157,0.870825679759361,0.35935316117294,0.266295577250848,0.180972314684754,0.210112825589136,0.125791643865697,0.0670836484804969,-0.234326584246244,0.114571823926554,0.0996329769991948,0.0920415610851054,0.0660362561562619,0.109730466394565,0.128341286079267,0.205820434083994,-0.307778238638146,-0.335613430323141,-0.309442564211598,0.268019156300213

"PHQ2.4",-0.0697988518767667,0.274582350132275,0.272006566679331,0.35935316117294,0.657987194278206,0.277511911691408,0.252926947435196,0.255588125603427,0.132466373652419,0.0893176439423777,-0.295911858711829,0.13694523933176,0.114916888286285,0.104606783477343,0.06681003684172,0.139900942130267,0.143775073805433,0.244146976504254,-0.365121661328045,-0.507284692038773,-0.445816181775722,0.331859548349094

"PHQ2.5",-0.0375597327319686,0.19248317375559,0.18261921276529,0.266295577250848,0.277511911691408,0.772958107792529,0.226452842946919,0.251060462943922,0.148297508217063,0.0903092682892824,-0.206498363558415,0.0909993970087451,0.122881252548771,0.0790406513699752,0.0565382725171922,0.116582956609028,0.111535954660634,0.154313478771571,-0.235501197268763,-0.299376444303475,-0.319303039703314,0.250223943283967

"PHQ2.6",-0.0740616166439528,0.228500573364517,0.297241576421299,0.180972314684754,0.252926947435196,0.226452842946919,0.628660209199625,0.223856146502476,0.143424781197826,0.152269233852556,-0.211991509325451,0.0593301568822912,0.0393443080065388,0.0613656880548478,0.0449873302126503,0.114139622102244,0.126901775164428,0.141206261349655,-0.320590931429786,-0.278154986179998,-0.432229708298624,0.272721093888875

"PHQ2.7",-0.0516446325064569,0.221071233134544,0.195289000115021,0.210112825589136,0.255588125603427,0.251060462943922,0.223856146502476,0.654653314883427,0.163783578421975,0.100727423554302,-0.198565368783178,0.0927595737932333,0.0502364910788664,0.0893612126746671,0.0531364259000429,0.114066426631998,0.15864595351042,0.200126872148426,-0.304329337790124,-0.34625291474819,-0.334517241018742,0.263806931262482

"PHQ2.8",-0.0365524236414398,0.105795338494195,0.103470510939237,0.125791643865697,0.132466373652419,0.148297508217063,0.143424781197826,0.163783578421975,0.378099915302384,0.0561304691829643,-0.113083516031551,0.0857781201311942,0.066834435331802,0.0567369459364315,0.0447294033174975,0.0463989571388239,0.0735405346057727,0.113679536289268,-0.139653471730864,-0.139249153895219,-0.151633130361133,0.136293451096712

"PHQ2.9",-0.0218697608599422,0.111785167809329,0.146173096830636,0.0670836484804969,0.0893176439423777,0.0903092682892824,0.152269233852556,0.100727423554302,0.0561304691829643,0.25988051710857,-0.106032352397849,0.0412299627400201,0.0291945361324211,0.0263677967814906,0.0251618142717225,0.0438615141702945,0.0469688361571681,0.0754593015757939,-0.126556710804697,-0.11886770093028,-0.225738315737375,0.126476544337285

"SF2.1",0.0388040557261513,-0.23748096046399,-0.258711132333925,-0.234326584246244,-0.295911858711829,-0.206498363558415,-0.211991509325451,-0.198565368783178,-0.113083516031551,-0.106032352397849,0.532165923674552,-0.176320916825547,-0.138342924263601,-0.128349999825725,-0.109123989641098,-0.130392501995448,-0.14976838861915,-0.341348818241705,0.387625782930119,0.470061309920077,0.448796283064311,-0.37060260785004

"SF2.2",-0.00976985252855495,0.0915396492891326,0.0895738280882389,0.114571823926554,0.13694523933176,0.0909993970087451,0.0593301568822912,0.0927595737932333,0.0857781201311942,0.0412299627400201,-0.176320916825547,0.359417642896728,0.203814529649394,0.134557672802306,0.150287727908039,0.0507941708521695,0.0758200506791494,0.366887066360408,-0.130434327978446,-0.19987591625044,-0.159123466816311,0.194968334245372

"SF2.3",-0.00189959672781393,0.0651788235048082,0.07494519053478,0.0996329769991948,0.114916888286285,0.122881252548771,0.0393443080065388,0.0502364910788664,0.066834435331802,0.0291945361324211,-0.138342924263601,0.203814529649394,0.351930791940133,0.116589927606194,0.105798823992778,0.0382289484599325,0.0468137314702182,0.284542162333611,-0.0731187892772122,-0.181301694300861,-0.106858415562054,0.129036643046605

"SF2.4",-0.0202124062836568,0.0739030264584197,0.0765833748688581,0.0920415610851054,0.104606783477343,0.0790406513699752,0.0613656880548478,0.0893612126746671,0.0567369459364315,0.0263677967814906,-0.128349999825725,0.134557672802306,0.116589927606194,0.244668058542434,0.128855397120281,0.0661861325953371,0.0638055370630492,0.304144606365218,-0.106157830346842,-0.169342948662091,-0.132400149179339,0.149127056879851

"SF2.5",-0.0077134083644995,0.067723237470504,0.0555239924294971,0.0660362561562619,0.06681003684172,0.0565382725171922,0.0449873302126503,0.0531364259000429,0.0447294033174975,0.0251618142717225,-0.109123989641098,0.150287727908039,0.105798823992778,0.128855397120281,0.206271806150511,0.0370891904232441,0.0513065391438918,0.278355402348529,-0.0821218321174752,-0.137607484062558,-0.102414404868544,0.132804467014984

"SF2.6",-0.0333631924378623,0.122964904514766,0.127875972018417,0.109730466394565,0.139900942130267,0.116582956609028,0.114139622102244,0.114066426631998,0.0463989571388239,0.0438615141702945,-0.130392501995448,0.0507941708521695,0.0382289484599325,0.0661861325953371,0.0370891904232441,0.215682652325002,0.124512465885683,0.11199604047361,-0.158799315448078,-0.216571454463704,-0.213608780668031,0.20233667825014

"SF2.7",-0.0204476774380191,0.134712777489256,0.132809695262859,0.128341286079267,0.143775073805433,0.111535954660634,0.126901775164428,0.15864595351042,0.0735405346057727,0.0469688361571681,-0.14976838861915,0.0758200506791494,0.0468137314702182,0.0638055370630492,0.0513065391438918,0.124512465885683,0.250302367002088,0.144210761128326,-0.193122762745597,-0.221968749019704,-0.235511653764513,0.209319874661471

"SF2.8",-0.0226783965312318,0.184639059194222,0.202882158778402,0.205820434083994,0.244146976504254,0.154313478771571,0.141206261349655,0.200126872148426,0.113679536289268,0.0754593015757939,-0.341348818241705,0.366887066360408,0.284542162333611,0.304144606365218,0.278355402348529,0.11199604047361,0.144210761128326,1.25728382066413,-0.295481399636811,-0.390492605514756,-0.356270237676148,0.338234525257666

"SF2.9",0.037244295110194,-0.310336594598174,-0.331619048946857,-0.307778238638146,-0.365121661328045,-0.235501197268763,-0.320590931429786,-0.304329337790124,-0.139653471730864,-0.126556710804697,0.387625782930119,-0.130434327978446,-0.0731187892772122,-0.106157830346842,-0.0821218321174752,-0.158799315448078,-0.193122762745597,-0.295481399636811,1.14803435307404,0.609894284827973,0.624298107722819,-0.453566187875345

"SF2.10",0.0779793170514076,-0.387550844710582,-0.368324834525955,-0.335613430323141,-0.507284692038773,-0.299376444303475,-0.278154986179998,-0.34625291474819,-0.139249153895219,-0.11886770093028,0.470061309920077,-0.19987591625044,-0.181301694300861,-0.169342948662091,-0.137607484062558,-0.216571454463704,-0.221968749019704,-0.390492605514756,0.609894284827973,1.21076635657348,0.679037862971109,-0.57364858506185

"SF2.11",0.0664231464989909,-0.43888701059243,-0.506282611196119,-0.309442564211598,-0.445816181775722,-0.319303039703314,-0.432229708298624,-0.334517241018742,-0.151633130361133,-0.225738315737375,0.448796283064311,-0.159123466816311,-0.106858415562054,-0.132400149179339,-0.102414404868544,-0.213608780668031,-0.235511653764513,-0.356270237676148,0.624298107722819,0.679037862971109,1.14081762825763,-0.628104272175613

"SF2.12",-0.0666514466561869,0.2993921290471,0.322758911548502,0.268019156300213,0.331859548349094,0.250223943283967,0.272721093888875,0.263806931262482,0.136293451096712,0.126476544337285,-0.37060260785004,0.194968334245372,0.129036643046605,0.149127056879851,0.132804467014984,0.20233667825014,0.209319874661471,0.338234525257666,-0.453566187875345,-0.57364858506185,-0.628104272175613,0.96668386179301

**4 Months**

"","Treatment","PHQ3.1","PHQ3.2","PHQ3.3","PHQ3.4","PHQ3.5","PHQ3.6","PHQ3.7","PHQ3.8","PHQ3.9"

"Treatment",0.250388840581485,-0.0517800938116175,-0.058509791434485,-0.0622083695638862,-0.0733837091105042,-0.0313705558889447,-0.0728570903702252,-0.0464710420927584,-0.0467343514628979,-0.0258349356422912

"PHQ3.1",-0.0517800938116175,0.531137863887426,0.363556758477949,0.152933756261252,0.31783278017954,0.198590376348695,0.19495915643026,0.274154042105005,0.120179295310644,0.0882698737339718

"PHQ3.2",-0.058509791434485,0.363556758477949,0.586172583983442,0.177877726476676,0.320808788409489,0.214257283871995,0.273125298519344,0.247134214297086,0.13437350740328,0.13409488934883

"PHQ3.3",-0.0622083695638862,0.152933756261252,0.177877726476676,0.845856857678224,0.317991990496369,0.27528994648084,0.130558583273119,0.160199257834985,0.0962977477863643,0.0456413114031328

"PHQ3.4",-0.0733837091105042,0.31783278017954,0.320808788409489,0.317991990496369,0.680660845284313,0.301127943859993,0.185822933633792,0.309321151702938,0.140803154813663,0.0838548491788422

"PHQ3.5",-0.0313705558889447,0.198590376348695,0.214257283871995,0.27528994648084,0.301127943859993,0.78618972970987,0.206881559771227,0.2162596597798,0.175817177568491,0.0666142089084296

"PHQ3.6",-0.0728570903702252,0.19495915643026,0.273125298519344,0.130558583273119,0.185822933633792,0.206881559771227,0.681383415183765,0.201835817422507,0.12480864144612,0.115739776860617

"PHQ3.7",-0.0464710420927584,0.274154042105005,0.247134214297086,0.160199257834985,0.309321151702938,0.2162596597798,0.201835817422507,0.702200164109096,0.183263321617087,0.0646853146853147

"PHQ3.8",-0.0467343514628979,0.120179295310644,0.13437350740328,0.0962977477863643,0.140803154813663,0.175817177568491,0.12480864144612,0.183263321617087,0.350630105446218,0.0627533587253377

"PHQ3.9",-0.0258349356422912,0.0882698737339718,0.13409488934883,0.0456413114031328,0.0838548491788422,0.0666142089084296,0.115739776860617,0.0646853146853147,0.0627533587253377,0.208378749096788

**5 Months**

"","Treatment","PHQ4.1","PHQ4.2","PHQ4.3","PHQ4.4","PHQ4.5","PHQ4.6","PHQ4.7","PHQ4.8","PHQ4.9"

"Treatment",0.250458322930413,-0.0589150051717373,-0.0623675856903378,-0.0961301137782216,-0.0707600670542497,-0.0296893390876342,-0.0578735242714984,-0.0453543531761601,-0.0340585654670614,-0.029211399222456

"PHQ4.1",-0.0589150051717373,0.529956842743518,0.357591753753968,0.206498555480258,0.316624460534294,0.180482933266755,0.265534828975996,0.280087027856047,0.114605699611228,0.149409708599351

"PHQ4.2",-0.0623675856903378,0.357591753753968,0.59355851196633,0.243578128901095,0.309890501836858,0.148781966686878,0.320633448657132,0.221157755822663,0.110618111780861,0.189891928523023

"PHQ4.3",-0.0961301137782216,0.206498555480258,0.243578128901095,0.842401112815208,0.347212611905696,0.231116025252345,0.189970396262082,0.219003459713949,0.101287584263652,0.113100545707458

"PHQ4.4",-0.0707600670542497,0.316624460534294,0.309890501836858,0.347212611905696,0.653853836002425,0.282326925134644,0.244869279880158,0.328794093519278,0.119381531547598,0.124906373720441

"PHQ4.5",-0.0296893390876342,0.180482933266755,0.148781966686878,0.231116025252345,0.282326925134644,0.738224489068017,0.190904875699968,0.201712023397653,0.130741520134109,0.0825337946285266

"PHQ4.6",-0.0578735242714984,0.265534828975996,0.320633448657132,0.189970396262082,0.244869279880158,0.190904875699968,0.643421193422977,0.239348004422727,0.102771337874951,0.202446766772479

"PHQ4.7",-0.0453543531761601,0.280087027856047,0.221157755822663,0.219003459713949,0.328794093519278,0.201712023397653,0.239348004422727,0.635146413667653,0.129521703463281,0.104825765952135

"PHQ4.8",-0.0340585654670614,0.114605699611228,0.110618111780861,0.101287584263652,0.119381531547598,0.130741520134109,0.102771337874951,0.129521703463281,0.275211327888148,0.0623461853978671

"PHQ4.9",-0.029211399222456,0.149409708599351,0.189891928523023,0.113100545707458,0.124906373720441,0.0825337946285266,0.202446766772479,0.104825765952135,0.0623461853978671,0.296465385026929

**6 Months**

"","Treatment","PHQ5.1","PHQ5.2","PHQ5.3","PHQ5.4","PHQ5.5","PHQ5.6","PHQ5.7","PHQ5.8","PHQ5.9","SF5.1","SF5.2","SF5.3","SF5.4","SF5.5","SF5.6","SF5.7","SF5.8","SF5.9","SF5.10","SF5.11","SF5.12"

"Treatment",0.250343878954608,-0.0426656208199287,-0.0441074493844283,-0.0713866184667735,-0.0486602930644446,-0.0476182356262386,-0.0511081807192849,-0.0551381010303106,-0.0397440706931766,-0.0294712790023532,0.0417694514230715,0.000992796541127165,-0.00867939113531211,-0.00933683464632571,-0.0128836192360014,-0.0147233242769069,-0.0107028772153194,-0.0109586549501518,0.0561365815211007,0.0695639652748569,0.06056627295842,-0.0284481680630236

"PHQ5.1",-0.0426656208199287,0.474128555784177,0.334138559535583,0.21283928442863,0.302439172265357,0.226033626246206,0.263811050356005,0.238538315504678,0.13016813123103,0.11366762535951,-0.25808920769531,0.0910530843005521,0.0891034895661631,0.0413981000450927,0.0557197585458183,0.141346565568149,0.127356470797761,0.170565856135445,-0.346241583017874,-0.421828640285562,-0.422755124080621,0.344411351226407

"PHQ5.2",-0.0441074493844283,0.334138559535583,0.549024065842873,0.217757795536963,0.309983668117968,0.22653760311632,0.340646681899652,0.238379164914116,0.129933194644962,0.155160457898985,-0.290942436747114,0.0897097775302102,0.0767503722987029,0.0525803236819868,0.0556136581521101,0.147238926718732,0.141462139211295,0.193962887598001,-0.403126551244596,-0.412919996513844,-0.550755775840183,0.363890246721308

"PHQ5.3",-0.0713866184667735,0.21283928442863,0.217757795536963,0.816321271992149,0.353988806408464,0.264053565541624,0.170368812547129,0.195939007430817,0.102636973713627,0.0918166282052739,-0.225357236236316,0.115333022610752,0.116837374621544,0.0528720997646845,0.0580823869557145,0.0968639755059662,0.0939746344273042,0.251812232617535,-0.301101549444678,-0.349494696874964,-0.254051708784734,0.255232075664738

"PHQ5.4",-0.0486602930644446,0.302439172265357,0.309983668117968,0.353988806408464,0.686420286395277,0.317577424867659,0.229309475901948,0.29089696514981,0.141606132602756,0.111386466894783,-0.331900978018272,0.179302086767386,0.1614620634253,0.103068953888011,0.0929212090897723,0.156949007392924,0.15108127669088,0.29463321472825,-0.407836650865287,-0.567116077620017,-0.453855801986351,0.35673794339544

"PHQ5.5",-0.0476182356262386,0.226033626246206,0.22653760311632,0.264053565541624,0.317577424867659,0.796704067055449,0.208629372378278,0.225573226323508,0.198396368335095,0.079054266562082,-0.224694108775639,0.123248869841342,0.134910439899811,0.06008313723707,0.0636052913782062,0.111623298130738,0.113389111826026,0.216346281370665,-0.324293958719368,-0.351641335197669,-0.313981379380904,0.265021731634211

"PHQ5.6",-0.0511081807192849,0.263811050356005,0.340646681899652,0.170368812547129,0.229309475901948,0.208629372378278,0.644931243155577,0.262810675215327,0.114772206244008,0.157811073091803,-0.229084012565318,0.0497440328001789,0.0432832766833017,0.0472771986464621,0.032415564927757,0.120528152602681,0.111339100647591,0.149285148597391,-0.394534314004115,-0.334648220355361,-0.468018309896514,0.313189415727868

"PHQ5.7",-0.0551381010303106,0.238538315504678,0.238379164914116,0.195939007430817,0.29089696514981,0.225573226323508,0.262810675215327,0.638205236054429,0.177452908477043,0.107629376167578,-0.238888825733893,0.0997627898340666,0.112246637943774,0.0571464299112167,0.0645886146698952,0.131344708811259,0.157320358770903,0.211946904331549,-0.335462919807049,-0.368729182534359,-0.328570183515788,0.270665893649512

"PHQ5.8",-0.0397440706931766,0.13016813123103,0.129933194644962,0.102636973713627,0.141606132602756,0.198396368335095,0.114772206244008,0.177452908477043,0.336334458755367,0.0784669250969113,-0.149641342776268,0.079054266562082,0.0740012353117267,0.0318206448630358,0.0494314155687171,0.0636261325269703,0.0779648428766848,0.124431131371234,-0.202737011227695,-0.181071689762449,-0.211780175141436,0.162574222909349

"PHQ5.9",-0.0294712790023532,0.11366762535951,0.155160457898985,0.0918166282052739,0.111386466894783,0.079054266562082,0.157811073091803,0.107629376167578,0.0784669250969113,0.225395129234069,-0.117129150704241,0.0431506511911664,0.0371540842967628,0.0180711706283796,0.0382359293826094,0.0538819481548005,0.0463185058033126,0.0946377618879807,-0.157104368683711,-0.16809712733184,-0.230408372836784,0.128536837677766

"SF5.1",0.0417694514230715,-0.25808920769531,-0.290942436747114,-0.225357236236316,-0.331900978018272,-0.224694108775639,-0.229084012565318,-0.238888825733893,-0.149641342776268,-0.117129150704241,0.542089647254084,-0.211304618019636,-0.173045952838375,-0.125422033262473,-0.114722945346929,-0.148013838522779,-0.155437076782581,-0.360263886836352,0.440951341601585,0.513434962353307,0.486595352044896,-0.403882516549767

"SF5.2",0.000992796541127165,0.0910530843005521,0.0897097775302102,0.115333022610752,0.179302086767386,0.123248869841342,0.0497440328001789,0.0997627898340666,0.079054266562082,0.0431506511911664,-0.211304618019636,0.398437292772669,0.238835775537038,0.141630763051296,0.155581070174043,0.0666992546447342,0.0778587424829766,0.416999177721949,-0.152632994948863,-0.226181408937442,-0.165916385311158,0.221024171943267

"SF5.3",-0.00867939113531211,0.0891034895661631,0.0767503722987029,0.116837374621544,0.1614620634253,0.134910439899811,0.0432832766833017,0.112246637943774,0.0740012353117267,0.0371540842967628,-0.173045952838375,0.238835775537038,0.380324439846761,0.111566458634109,0.114775995543783,0.0582907984433556,0.0636734987741615,0.324460687909481,-0.117743017267839,-0.198330055589028,-0.142291995862085,0.166181636295429

"SF5.4",-0.00933683464632571,0.0413981000450927,0.0525803236819868,0.0528720997646845,0.103068953888011,0.06008313723707,0.0472771986464621,0.0571464299112167,0.0318206448630358,0.0180711706283796,-0.125422033262473,0.141630763051296,0.111566458634109,0.247081291848079,0.123497068976624,0.0658428728955176,0.0620744142689872,0.255720895335751,-0.0941072599194395,-0.151810716897624,-0.091456644726621,0.119518304212565

"SF5.5",-0.0128836192360014,0.0557197585458183,0.0556136581521101,0.0580823869557145,0.0929212090897723,0.0636052913782062,0.032415564927757,0.0645886146698952,0.0494314155687171,0.0382359293826094,-0.114722945346929,0.155581070174043,0.114775995543783,0.123497068976624,0.207430058999398,0.0324667204747235,0.0484064099794999,0.26480574154702,-0.0877374469971694,-0.113588050064229,-0.0918658891023528,0.114787363443109

"SF5.6",-0.0147233242769069,0.141346565568149,0.147238926718732,0.0968639755059662,0.156949007392924,0.111623298130738,0.120528152602681,0.131344708811259,0.0636261325269703,0.0538819481548005,-0.148013838522779,0.0666992546447342,0.0582907984433556,0.0658428728955176,0.0324667204747235,0.235504981034555,0.135456099067453,0.1325193917416,-0.222676306645295,-0.275631771005036,-0.247778523006734,0.230918033656561

"SF5.7",-0.0107028772153194,0.127356470797761,0.141462139211295,0.0939746344273042,0.15108127669088,0.113389111826026,0.111339100647591,0.157320358770903,0.0779648428766848,0.0463185058033126,-0.155437076782581,0.0778587424829766,0.0636734987741615,0.0620744142689872,0.0484064099794999,0.135456099067453,0.248695533552355,0.139940735351514,-0.237528467114562,-0.241456076331655,-0.233752429888481,0.219629709625958

"SF5.8",-0.0109586549501518,0.170565856135445,0.193962887598001,0.251812232617535,0.29463321472825,0.216346281370665,0.149285148597391,0.211946904331549,0.124431131371234,0.0946377618879807,-0.360263886836352,0.416999177721949,0.324460687909481,0.255720895335751,0.26480574154702,0.1325193917416,0.139940735351514,1.22364447273788,-0.324600892001167,-0.375261935346967,-0.355788723801729,0.381034933554628

"SF5.9",0.0561365815211007,-0.346241583017874,-0.403126551244596,-0.301101549444678,-0.407836650865287,-0.324293958719368,-0.394534314004115,-0.335462919807049,-0.202737011227695,-0.157104368683711,0.440951341601585,-0.152632994948863,-0.117743017267839,-0.0941072599194395,-0.0877374469971694,-0.222676306645295,-0.237528467114562,-0.324600892001167,1.2640118832441,0.738909666882657,0.741643646670532,-0.548484090624893

"SF5.10",0.0695639652748569,-0.421828640285562,-0.412919996513844,-0.349494696874964,-0.567116077620017,-0.351641335197669,-0.334648220355361,-0.368729182534359,-0.181071689762449,-0.16809712733184,0.513434962353307,-0.226181408937442,-0.198330055589028,-0.151810716897624,-0.113588050064229,-0.275631771005036,-0.241456076331655,-0.375261935346967,0.738909666882657,1.32915373568118,0.719406140939216,-0.63425110173891

"SF5.11",0.06056627295842,-0.422755124080621,-0.550755775840183,-0.254051708784734,-0.453855801986351,-0.313981379380904,-0.468018309896514,-0.328570183515788,-0.211780175141436,-0.230408372836784,0.486595352044896,-0.165916385311158,-0.142291995862085,-0.091456644726621,-0.0918658891023528,-0.247778523006734,-0.233752429888481,-0.355788723801729,0.741643646670532,0.719406140939216,1.19225012409957,-0.619673665503352

"SF5.12",-0.0284481680630236,0.344411351226407,0.363890246721308,0.255232075664738,0.35673794339544,0.265021731634211,0.313189415727868,0.270665893649512,0.162574222909349,0.128536837677766,-0.403882516549767,0.221024171943267,0.166181636295429,0.119518304212565,0.114787363443109,0.230918033656561,0.219629709625958,0.381034933554628,-0.548484090624893,-0.63425110173891,-0.619673665503352,1.02010602460771

**7 Months**

"","Treatment","PHQ6.1","PHQ6.2","PHQ6.3","PHQ6.4","PHQ6.5","PHQ6.6","PHQ6.7","PHQ6.8","PHQ6.9"

"Treatment",0.250143893173708,-0.0469820805034342,-0.0268715705460266,-0.0662752772341813,-0.0563984497908753,-0.0256475192816853,-0.056590307355819,-0.0181573999462799,-0.0290280495759948,-0.0194466827827021

"PHQ6.1",-0.0469820805034342,0.559518053796861,0.367890717931008,0.220993054756149,0.356064617627873,0.205537009324278,0.304328306665132,0.293714746172442,0.130923602317639,0.124791834542036

"PHQ6.2",-0.0268715705460266,0.367890717931008,0.570123939986954,0.216242661448141,0.322209431717893,0.219941675300257,0.316208127086451,0.24842868654311,0.127688883772687,0.14590384098845

"PHQ6.3",-0.0662752772341813,0.220993054756149,0.216242661448141,0.85784889298185,0.391615824411957,0.278630904416561,0.191093971835309,0.193043244695138,0.0912397835846667,0.0733011012624228

"PHQ6.4",-0.0563984497908753,0.356064617627873,0.322209431717893,0.391615824411957,0.701837995472162,0.293008710333448,0.229323510226008,0.316680096696213,0.131303480296228,0.0704807950577491

"PHQ6.5",-0.0256475192816853,0.205537009324278,0.219941675300257,0.278630904416561,0.293008710333448,0.73894324853229,0.172303441924715,0.195433789954338,0.117881125052761,0.0522159548751007

"PHQ6.6",-0.056590307355819,0.304328306665132,0.316208127086451,0.191093971835309,0.229323510226008,0.172303441924715,0.642032155327885,0.204942250872952,0.111837611757032,0.152618855761483

"PHQ6.7",-0.0181573999462799,0.293714746172442,0.24842868654311,0.193043244695138,0.316680096696213,0.195433789954338,0.204942250872952,0.642385173247381,0.152887456352404,0.0628793983346763

"PHQ6.8",-0.0290280495759948,0.130923602317639,0.127688883772687,0.0912397835846667,0.131303480296228,0.117881125052761,0.111837611757032,0.152887456352404,0.30559840374506,0.0298415256513564

"PHQ6.9",-0.0194466827827021,0.124791834542036,0.14590384098845,0.0733011012624228,0.0704807950577491,0.0522159548751007,0.152618855761483,0.0628793983346763,0.0298415256513564,0.249905989793178

**8 Months**

"","Treatment","PHQ7.1","PHQ7.2","PHQ7.3","PHQ7.4","PHQ7.5","PHQ7.6","PHQ7.7","PHQ7.8","PHQ7.9"

"Treatment",0.250353321219463,-0.0420568730683812,-0.0300517169081675,-0.0335616331982171,-0.0487816241904673,-0.00689558775567255,-0.0733820994269207,-0.0350991629004954,-0.028110391526503,-0.0251440463433195

"PHQ7.1",-0.0420568730683812,0.525753622513162,0.375918246905527,0.246625976486667,0.357654257714827,0.258856326391154,0.254461165727065,0.307175915140785,0.113668483747224,0.108225007377036

"PHQ7.2",-0.0300517169081675,0.375918246905527,0.566494276972775,0.257621643448415,0.33640839273789,0.243286896830204,0.331329885539456,0.282082343257389,0.0982776561213872,0.154187827113327

"PHQ7.3",-0.0335616331982171,0.246625976486667,0.257621643448415,0.864775039214773,0.413222755439594,0.380453182997096,0.196710618273308,0.243131590799671,0.116479522899874,0.0945658419916445

"PHQ7.4",-0.0487816241904673,0.357654257714827,0.33640839273789,0.413222755439594,0.697106648651167,0.366615415676591,0.269331718150616,0.348025283821771,0.140163692556182,0.0886331516252776

"PHQ7.5",-0.00689558775567255,0.258856326391154,0.243286896830204,0.380453182997096,0.366615415676591,0.813663824566308,0.237804593952383,0.240499153582134,0.18192548416655,0.0764571588314774

"PHQ7.6",-0.0733820994269207,0.254461165727065,0.331329885539456,0.196710618273308,0.269331718150616,0.237804593952383,0.677553619407042,0.242564723788225,0.100032614266412,0.130736616502819

"PHQ7.7",-0.0350991629004954,0.307175915140785,0.282082343257389,0.243131590799671,0.348025283821771,0.240499153582134,0.242564723788225,0.678046716053984,0.176101508021556,0.0832673282703567

"PHQ7.8",-0.028110391526503,0.113668483747224,0.0982776561213872,0.116479522899874,0.140163692556182,0.18192548416655,0.100032614266412,0.176101508021556,0.305486962058737,0.0258739846868254

"PHQ7.9",-0.0251440463433195,0.108225007377036,0.154187827113327,0.0945658419916445,0.0886331516252776,0.0764571588314774,0.130736616502819,0.0832673282703567,0.0258739846868254,0.256177297364457

**9 Months**

"","Treatment","PHQ8.1","PHQ8.2","PHQ8.3","PHQ8.4","PHQ8.5","PHQ8.6","PHQ8.7","PHQ8.8","PHQ8.9"

"Treatment",0.250244438518662,-0.0214863478057098,-0.0208277779124546,-0.0398737808376363,-0.0442211501943387,-0.0200439585302983,-0.0340597803689608,-0.0301770462130211,-0.0198136610829636,-0.011696686140942

"PHQ8.1",-0.0214863478057098,0.509450275952906,0.358407472950151,0.195760910846606,0.339426114922467,0.180391586466591,0.237060919735279,0.262062333842412,0.107884253311031,0.0860827616300211

"PHQ8.2",-0.0208277779124546,0.358407472950151,0.529898265092563,0.200750688872189,0.293176731069146,0.181720847171382,0.294223170347386,0.254931193587226,0.113201296130195,0.119063780271993

"PHQ8.3",-0.0398737808376363,0.195760910846606,0.200750688872189,0.814885295710003,0.354831802057324,0.244499123253578,0.139317834719158,0.214075618368848,0.0987208390907695,0.0639620857676177

"PHQ8.4",-0.0442211501943387,0.339426114922467,0.293176731069146,0.354831802057324,0.675058382423052,0.25893109661988,0.214952364791157,0.305378455471787,0.128279718471471,0.0782647693389251

"PHQ8.5",-0.0200439585302983,0.180391586466591,0.181720847171382,0.244499123253578,0.25893109661988,0.652658925440191,0.169159535526411,0.21395440918604,0.111641737978069,0.050677559331895

"PHQ8.6",-0.0340597803689608,0.237060919735279,0.294223170347386,0.139317834719158,0.214952364791157,0.169159535526411,0.613851785411263,0.228430825919372,0.121306150153936,0.129378681728928

"PHQ8.7",-0.0301770462130211,0.262062333842412,0.254931193587226,0.214075618368848,0.305378455471787,0.21395440918604,0.228430825919372,0.658089096829976,0.145556067327661,0.0513805725921796

"PHQ8.8",-0.0198136610829636,0.107884253311031,0.113201296130195,0.0987208390907695,0.128279718471471,0.111641737978069,0.121306150153936,0.145556067327661,0.301697736620526,0.0276599355167147

"PHQ8.9",-0.011696686140942,0.0860827616300211,0.119063780271993,0.0639620857676177,0.0782647693389251,0.050677559331895,0.129378681728928,0.0513805725921796,0.0276599355167147,0.223061259120991

**10 Months**

"","Treatment","PHQ9.1","PHQ9.2","PHQ9.3","PHQ9.4","PHQ9.5","PHQ9.6","PHQ9.7","PHQ9.8","PHQ9.9"

"Treatment",0.24983344437042,-0.0176548967355097,-0.0200977126360204,-0.0253164556962025,-0.0278703086831002,-0.0154341550077726,-0.0450810570730624,-0.0142127470575172,-0.0068842993559849,-0.0273151232511659

"PHQ9.1",-0.0176548967355097,0.575838330002221,0.386942038640906,0.264556962025316,0.386675549633578,0.276215856095936,0.26866533422163,0.308216744392627,0.154652453919609,0.118099045081057

"PHQ9.2",-0.0200977126360204,0.386942038640906,0.548096824339329,0.260506329113924,0.344690206528981,0.227372862536087,0.321709971130358,0.257934710193205,0.160799467021985,0.145494115034422

"PHQ9.3",-0.0253164556962025,0.264556962025316,0.260506329113924,0.796118143459916,0.377299578059072,0.306244725738397,0.189789029535865,0.210717299578059,0.0980590717299578,0.113839662447257

"PHQ9.4",-0.0278703086831002,0.386675549633578,0.344690206528981,0.377299578059072,0.681572285143238,0.346409060626249,0.293089051743282,0.337739284921164,0.16192316233622,0.115429713524317

"PHQ9.5",-0.0154341550077726,0.276215856095936,0.227372862536087,0.306244725738397,0.346409060626249,0.708221185876083,0.215793915167666,0.24964690206529,0.140986009327115,0.0990939373750833

"PHQ9.6",-0.0450810570730624,0.26866533422163,0.321709971130358,0.189789029535865,0.293089051743282,0.215793915167666,0.644530313124584,0.250157672662669,0.152711525649567,0.152191872085276

"PHQ9.7",-0.0142127470575172,0.308216744392627,0.257934710193205,0.210717299578059,0.337739284921164,0.24964690206529,0.250157672662669,0.685294248278925,0.204747945813902,0.094594714634688

"PHQ9.8",-0.0068842993559849,0.154652453919609,0.160799467021985,0.0980590717299578,0.16192316233622,0.140986009327115,0.152711525649567,0.204747945813902,0.393311125916056,0.0403419942260715

"PHQ9.9",-0.0273151232511659,0.118099045081057,0.145494115034422,0.113839662447257,0.115429713524317,0.0990939373750833,0.152191872085276,0.094594714634688,0.0403419942260715,0.255678436597824

**11 Months.**

"","Treatment","PHQ10.1","PHQ10.2","PHQ10.3","PHQ10.4","PHQ10.5","PHQ10.6","PHQ10.7","PHQ10.8","PHQ10.9"

"Treatment",0.250170374651702,-0.0137852502997731,0.00499478092839089,-0.0181244123152837,-0.0278206709741979,-0.0167355354077346,-0.026759603523089,-0.0127931953658095,-0.00888536158245702,-0.0169857057823863

"PHQ10.1",-0.0137852502997731,0.546566195943789,0.41275523848138,0.289322038284694,0.410447632439334,0.251132236609415,0.310094805945428,0.321188568076535,0.147859317983799,0.133936042649736

"PHQ10.2",0.00499478092839089,0.41275523848138,0.64423184755135,0.297073006616575,0.428455586131935,0.289999223609182,0.381639219813494,0.32573045436116,0.176076810931583,0.156977596811622

"PHQ10.3",-0.0181244123152837,0.289322038284694,0.297073006616575,0.879292794230554,0.443608146927649,0.352718661847293,0.220913380664418,0.267816012629291,0.116148066355535,0.0742315887544103

"PHQ10.4",-0.0278206709741979,0.410447632439334,0.428455586131935,0.443608146927649,0.759124748751305,0.35500470147773,0.297301610579619,0.370325480283986,0.158866814468474,0.118598010714193

"PHQ10.5",-0.0167355354077346,0.251132236609415,0.289999223609182,0.352718661847293,0.35500470147773,0.754069581870412,0.193618930133453,0.261898189284081,0.158297461202026,0.0912172945367966

"PHQ10.6",-0.026759603523089,0.310094805945428,0.381639219813494,0.220913380664418,0.297301610579619,0.193618930133453,0.668476807480957,0.261492740745853,0.127793928623804,0.141001199092485

"PHQ10.7",-0.0127931953658095,0.321188568076535,0.32573045436116,0.267816012629291,0.370325480283986,0.261898189284081,0.261492740745853,0.670391904831739,0.211230061852468,0.0875596311280959

"PHQ10.8",-0.00888536158245702,0.147859317983799,0.176076810931583,0.116148066355535,0.158866814468474,0.158297461202026,0.127793928623804,0.211230061852468,0.34049050646561,0.0318406500979115

"PHQ10.9",-0.0169857057823863,0.133936042649736,0.156977596811622,0.0742315887544103,0.118598010714193,0.0912172945367966,0.141001199092485,0.0875596311280959,0.0318406500979115,0.220529498537797

**12 Months**

"","Treatment","PHQ11.1","PHQ11.2","PHQ11.3","PHQ11.4","PHQ11.5","PHQ11.6","PHQ11.7","PHQ11.8","PHQ11.9","SF11.1","SF11.2","SF11.3","SF11.4","SF11.5","SF11.6","SF11.7","SF11.8","SF11.9","SF11.10","SF11.11","SF11.12"

"Treatment",0.25038752801556,-0.01116919917459,-0.0270998094448229,-0.0135733538698498,-0.0330904495324981,0.000397401340797567,-0.0250560311207211,-0.0152147941905354,-0.0111519208554249,-0.0231183910429193,-0.00223877649753661,-0.00771106701025838,-0.00542292388653575,-0.00134770889487871,-0.0102954098910972,-0.00726676737458409,0.000276453106641786,-0.00473919611385919,-0.00410730329867796,0.0285166316163621,0.0290991578053573,-0.0332286760858189

"PHQ11.1",-0.01116919917459,0.47320873196884,0.341340600100708,0.210338852522141,0.327564843063495,0.232307001174926,0.292800864903291,0.281162682779933,0.114700887611939,0.12689197594858,-0.274266165101745,0.11710010564458,0.0977409831857271,0.0831087151841869,0.0538713308255087,0.15686245470612,0.150666943119773,0.144002448584659,-0.348992427159543,-0.454392642398033,-0.435208771462141,0.36543891867342

"PHQ11.2",-0.0270998094448229,0.341340600100708,0.566511655460443,0.250770119368502,0.346380932634302,0.217793213076232,0.35999624813641,0.291235942853194,0.129180119072302,0.158459465063239,-0.322731850359883,0.115907901622187,0.0860608394301117,0.100404312668464,0.058398250446768,0.165968128906134,0.168325385306517,0.176191957189262,-0.427384161211654,-0.469960407965799,-0.555863274192115,0.386432076459031

"PHQ11.3",-0.0135733538698498,0.210338852522141,0.250770119368502,0.845591066615325,0.403670902323193,0.307726864330638,0.187588242844308,0.266300859966628,0.118919265819535,0.105570530098832,-0.271884225388269,0.163586189192658,0.155179052753177,0.105570530098832,0.089590553202413,0.10794506481838,0.117475292003594,0.228725452445129,-0.376299576434347,-0.390257989988448,-0.321909896033885,0.328423822359132

"PHQ11.4",-0.0330904495324981,0.327564843063495,0.346380932634302,0.403670902323193,0.680069705676175,0.3188886585113,0.286244976945786,0.348622177463148,0.158150923649576,0.136515999723547,-0.327772182893477,0.171926680686789,0.155820818893595,0.130952380952381,0.0956404332415114,0.174508555236318,0.158874144723201,0.259147635832272,-0.421736619175972,-0.574800311997077,-0.452687025463306,0.38600999180514

"PHQ11.5",0.000397401340797567,0.232307001174926,0.217793213076232,0.307726864330638,0.3188886585113,0.776245766811805,0.194191029096689,0.263580758863778,0.123453590434723,0.0735365263667151,-0.213318128412468,0.103307070288202,0.109389038634322,0.0943396226415094,0.0780634459879743,0.126546409565277,0.108974358974359,0.188696523602184,-0.292228212039533,-0.365471006980441,-0.288530651738199,0.266759969590158

"PHQ11.6",-0.0250560311207211,0.292800864903291,0.35999624813641,0.187588242844308,0.286244976945786,0.194191029096689,0.640364128234748,0.302229890504823,0.129313408963005,0.19118460156196,-0.256723734486538,0.0762220708312353,0.0423886535746374,0.0664869721473495,0.0216373922573384,0.140452988161883,0.140490013131523,0.0978890830642852,-0.409607239122064,-0.391753798761885,-0.497057749079312,0.331566008115873

"PHQ11.7",-0.0152147941905354,0.281162682779933,0.291235942853194,0.266300859966628,0.348622177463148,0.263580758863778,0.302229890504823,0.65435463009587,0.184542322008629,0.134960950998687,-0.270743856323371,0.119163630619156,0.107784623283276,0.0835579514824798,0.0528395683382206,0.158674209887148,0.174200013822655,0.173417552797607,-0.384610447952766,-0.421652695911456,-0.411394310989998,0.318125944136726

"PHQ11.8",-0.0111519208554249,0.114700887611939,0.129180119072302,0.118919265819535,0.158150923649576,0.123453590434723,0.129313408963005,0.184542322008629,0.308200783942024,0.0672644965097795,-0.136972641015768,0.102655430822547,0.0698883326915672,0.0595238095238095,0.0532320330163996,0.0749977785018216,0.0834197249291589,0.151933197081445,-0.165704017456039,-0.175710632583948,-0.183671001056446,0.174617161813927

"PHQ11.9",-0.0231183910429193,0.12689197594858,0.158459465063239,0.105570530098832,0.136515999723547,0.0735365263667151,0.19118460156196,0.134960950998687,0.0672644965097795,0.224963715529753,-0.101250950307554,0.0423837169120188,0.0231356693620845,0.0172955974842767,0.0141855000345566,0.0600939940562582,0.0506773101112724,0.041174234570461,-0.158131176999102,-0.161120326214666,-0.222631142442463,0.149699357246527

"SF11.1",-0.00223877649753661,-0.274266165101745,-0.322731850359883,-0.271884225388269,-0.327772182893477,-0.213318128412468,-0.256723734486538,-0.270743856323371,-0.136972641015768,-0.101250950307554,0.592982040421393,-0.21620854437566,-0.186914388396868,-0.16015274034142,-0.128530947937956,-0.169181896270845,-0.174372797014306,-0.34228843932348,0.448947503529714,0.544637303397411,0.490326609598847,-0.402535469920915

"SF11.2",-0.00771106701025838,0.11710010564458,0.115907901622187,0.163586189192658,0.171926680686789,0.103307070288202,0.0762220708312353,0.119163630619156,0.102655430822547,0.0423837169120188,-0.21620854437566,0.362444832795237,0.21864972404056,0.154986522911051,0.158400225111815,0.0642358539932664,0.0802059575644481,0.395256360889784,-0.156815556411244,-0.232761174135837,-0.173257111262502,0.199433764797646

"SF11.3",-0.00542292388653575,0.0977409831857271,0.0860608394301117,0.155179052753177,0.155820818893595,0.109389038634322,0.0423886535746374,0.107784623283276,0.0698883326915672,0.0231356693620845,-0.186914388396868,0.21864972404056,0.355410088563727,0.133198562443845,0.128096521627519,0.0549993582338596,0.0653638814016173,0.339943524579643,-0.112405339494288,-0.184443588756257,-0.125946605057117,0.171094853035554

"SF11.4",-0.00134770889487871,0.0831087151841869,0.100404312668464,0.105570530098832,0.130952380952381,0.0943396226415094,0.0664869721473495,0.0835579514824798,0.0595238095238095,0.0172955974842767,-0.16015274034142,0.154986522911051,0.133198562443845,0.245283018867925,0.130053908355795,0.0772686433063792,0.0754716981132075,0.263477088948787,-0.136118598382749,-0.183737646001797,-0.129380053908356,0.157681940700809

"SF11.5",-0.0102954098910972,0.0538713308255087,0.058398250446768,0.089590553202413,0.0956404332415114,0.0780634459879743,0.0216373922573384,0.0528395683382206,0.0532320330163996,0.0141855000345566,-0.128530947937956,0.158400225111815,0.128096521627519,0.130053908355795,0.210894227066734,0.0323474818083982,0.0463750086391596,0.271711442196617,-0.0780387626748813,-0.112392997837742,-0.0784657839913905,0.124339721374762

"SF11.6",-0.00726676737458409,0.15686245470612,0.165968128906134,0.10794506481838,0.174508555236318,0.126546409565277,0.140452988161883,0.158674209887148,0.0749977785018216,0.0600939940562582,-0.169181896270845,0.0642358539932664,0.0549993582338596,0.0772686433063792,0.0323474818083982,0.243061520689553,0.141423042366439,0.0794234965393995,-0.230747015787447,-0.306544533633482,-0.270057660219385,0.257098920845552

"SF11.7",0.000276453106641786,0.150666943119773,0.168325385306517,0.117475292003594,0.158874144723201,0.108974358974359,0.140490013131523,0.174200013822655,0.0834197249291589,0.0506773101112724,-0.174372797014306,0.0802059575644481,0.0653638814016173,0.0754716981132075,0.0463750086391596,0.141423042366439,0.247943880019352,0.0875837998479508,-0.213611859838275,-0.24077337756583,-0.245835925081208,0.233015412260695

"SF11.8",-0.00473919611385919,0.144002448584659,0.176191957189262,0.228725452445129,0.259147635832272,0.188696523602184,0.0978890830642852,0.173417552797607,0.151933197081445,0.041174234570461,-0.34228843932348,0.395256360889784,0.339943524579643,0.263477088948787,0.271711442196617,0.0794234965393995,0.0875837998479508,1.19802434762003,-0.234982672314209,-0.329329699949646,-0.27354047569681,0.332494100688171

"SF11.9",-0.00410730329867796,-0.348992427159543,-0.427384161211654,-0.376299576434347,-0.421736619175972,-0.292228212039533,-0.409607239122064,-0.384610447952766,-0.165704017456039,-0.158131176999102,0.448947503529714,-0.156815556411244,-0.112405339494288,-0.136118598382749,-0.0780387626748813,-0.230747015787447,-0.213611859838275,-0.234982672314209,1.2325118726736,0.757348222307791,0.676453600308048,-0.554710563470671

"SF11.10",0.0285166316163621,-0.454392642398033,-0.469960407965799,-0.390257989988448,-0.574800311997077,-0.365471006980441,-0.391753798761885,-0.421652695911456,-0.175710632583948,-0.161120326214666,0.544637303397411,-0.232761174135837,-0.184443588756257,-0.183737646001797,-0.112392997837742,-0.306544533633482,-0.24077337756583,-0.329329699949646,0.757348222307791,1.34704244542519,0.719696296515704,-0.631300415666992

"SF11.11",0.0290991578053573,-0.435208771462141,-0.555863274192115,-0.321909896033885,-0.452687025463306,-0.288530651738199,-0.497057749079312,-0.411394310989998,-0.183671001056446,-0.222631142442463,0.490326609598847,-0.173257111262502,-0.125946605057117,-0.129380053908356,-0.0784657839913905,-0.270057660219385,-0.245835925081208,-0.27354047569681,0.676453600308048,0.719696296515704,1.13362558376035,-0.644234471727733

"SF11.12",-0.0332286760858189,0.36543891867342,0.386432076459031,0.328423822359132,0.38600999180514,0.266759969590158,0.331566008115873,0.318125944136726,0.174617161813927,0.149699357246527,-0.402535469920915,0.199433764797646,0.171094853035554,0.157681940700809,0.124339721374762,0.257098920845552,0.233015412260695,0.332494100688171,-0.554710563470671,-0.631300415666992,-0.644234471727733,1.00849599636662
